# Effectiveness and safety of acupuncture therapies for intractable hiccups: a systematic review and network meta-analysis

**DOI:** 10.3389/fmed.2025.1676850

**Published:** 2025-11-19

**Authors:** Yan Zhai, You-Yi Hui, Ze-Fei Jiang, Guang Liu, Lin Ding, Jie Cheng, Xing Tang, Xue-Mei Li, Han Zhai, Ru-Long Ma, Zhao-Xin Wan, Hong Zhang

**Affiliations:** 1 School of Acupuncture-Moxibustion and Tuina, Chengdu University of Traditional Chinese Medicine, Chengdu, China; 2 Department of Hepatopathy, Xi'an Daxing Hospital, Xi'an, China; 3 Department of Rehabilitation, The Third Affiliated Hospital of Chengdu University of Traditional Chinese Medicine, Chengdu, China; 4 Sixth Department of Obstetrics (Foetal Protection Centre), Northwest Women's and Children's Hospital, Xi'an, China; 5 Department of Rehabilitation, Shaanxi Provincial Hospital of Traditional Chinese Medicine, Xi'an, China

**Keywords:** intractable hiccups, acupuncture therapy, effectiveness, network meta-analysis, systematic review, randomized controlled trials

## Abstract

**Background:**

Intractable hiccups (IH) have diverse causes, including central lesions (e.g., stroke, intracranial injury) and peripheral triggers (e.g., gastrointestinal disease, tumors, chemotherapy, cirrhosis, surgery). IH significantly impairs quality of life, delays recovery, and increases the risk of complications. Acupuncture is frequently used as a complementary or alternative therapy for IH in China, but no prior study has systematically compared different acupuncture modalities within a unified framework.

**Objective:**

To assess the comparative effectiveness and safety of multiple acupuncture therapies for treating IH via network meta-analysis.

**Methods:**

RCTs on acupuncture for IH published from January 2015 to January 2025 were retrieved from eight databases and four clinical trial registries. Study quality was assessed via the Cochrane risk of bias tool. Meta-analysis was performed with Stata software.

**Results:**

The 41 included studies were all conducted in China and published in Chinese and no studies or data from other countries. The studies covered a total of 3,060 patients with IH and 15 types of acupuncture-related intervention measures. Manual acupuncture combined with acupoint injection showed the highest efficacy in improving both total effective rate and quality of life. Electroacupuncture combined with conventional medicine was most effective in reducing hiccup symptoms. Among monotherapies, auricular acupressure achieved the greatest improvement in total effective rate. All acupuncture therapies demonstrated acceptable safety, with no serious adverse events reported.

**Conclusion:**

Acupuncture appears effective and safe for treating IH in China. Combination therapies outperformed monotherapies, with manual acupuncture plus acupoint injection showing the greatest efficacy and acceptable safety. Electroacupuncture with conventional medication was most effective for symptom relief. However, limitations of linguistic and geographical scope, potential favorable bias and uneven methodological quality warrant further high-quality international studies.

**Systematic review registration:**

https://www.crd.york.ac.uk/prospero/, identifier: CRD42025638314.

## 1 Introduction

Hiccups are a common but frequently underestimated symptom. Most people experience occasional hiccups, especially after overeating or consuming cold drinks. These episodes typically last from a few minutes to several hours and are considered acute and self-limiting, requiring no medical intervention. However, when hiccups persist for more than 48 h or occur in patients with underlying conditions, they may indicate a potential pathological process (1). This condition is referred to as intractable hiccups (IH) and is most commonly associated with gastrointestinal disorders (62.5%) and central nervous system diseases (33.3%) (2, 3). From a mechanistic perspective, the “hiccup reflex arc” involves afferent, central, and efferent components (4, 5). The afferent pathway consists of the phrenic nerve, vagus nerve, and sympathetic fibers arising from T6 to T12 (6, 7). The efferent pathway includes the phrenic nerve, intercostal nerves, the anterior scalene muscle, and the recurrent laryngeal branch of the vagus nerve (3). The effectors involved are the diaphragm, intercostal muscles, and anterior scalene muscles (8). The central nervous system regions implicated in the hiccup response include the cervical spinal cord segments (C3–C5), the medullary brainstem near the respiratory centers, the reticular formation, and the hypothalamus (5, 9, 10). The efferent response of the reflex is transmitted via the phrenic nerve to the diaphragm, resulting in diaphragmatic contraction and the characteristic hiccup.

Any disturbance affecting the afferent, central, or efferent components of the reflex arc can lead to hiccups. Owing to the long trajectory of the afferent and efferent nerves and the diffuse central process of the hiccup reflex arc (11) and considering the participation of anatomical structures with different main functions, it is not surprising that hiccups are a complication of a large number of various underlying diseases. Central causes include conditions such as stroke, brain tumors, and intracranial injury (4). Peripheral neurological disorders, such as gastrointestinal neurosis, gastritis, liver cirrhosis, and fulminant hepatitis, may also disrupt neurotransmitter balance within the hiccup reflex arc (3, 12). Additionally, ~3.9%−4.5% of cancer patients receiving chemotherapy, as well as some perioperative patients, experience hiccups (13). This may be associated with factors such as preoperative anxiety, the use of sedatives or anesthetics during surgery, the presence of tumors located near the diaphragm or phrenic nerve, and oropharyngeal intubation (14, 15).

The potential hazards of IH must not be underestimated. When incessant hiccups fail to resolve and interfere with oral intake, they can significantly disrupt a patient's nutritional status, emotional wellbeing, and sleep quality (16–18). Persistent hiccups may also delay recovery, prolong hospitalization, and increase the risk of aspiration pneumonia, respiratory distress and malnutrition (16, 17, 19, 20). Perhaps due to the prevalence of IH in both internal medicine and surgery, as well as in oncology and intense care units, and because it often complicates existing treatments, in the absence of agreed-upon management guidelines, chlorpromazine is currently the only FDA-approved medication for hiccups, while other agents such as baclofen and metoclopramide are used off-label, often limited by adverse effects including sedation and extrapyramidal symptoms (4).

Against this backdrop, acupuncture—a nonpharmacological procedure rooted in traditional Chinese medicine (TCM)—has garnered considerable attention for its promising potential in treating hiccups. Several randomized controlled trials have demonstrated its effectiveness in reducing the duration and frequency of hiccup episodes (21–24), and it appears to avoid the above adverse effects of conventional drugs (25, 26). Moretto et al. (1) compared different acupuncture techniques for persistent and intractable hiccups, but included only four studies—likely due to limited access to Chinese databases—and could not perform a meta-analysis due to methodological heterogeneity. In contrast, three meta-analyses confirmed the beneficial effects of acupuncture in cancer-related and poststroke hiccups (14, 27, 28). However, these studies are limited to specific populations and often include cases of acute hiccups lasting < 48 h. As a result, they do not offer a comprehensive assessment of IH across diverse etiologies. The heterogeneity of acupuncture and its technical variants, such as electroacupuncture (EA), transcutaneous electrical acupoint stimulation (TEAS), acupoint injection (AI), auricular acupressure (AA), etc. further complicates clinical decision-making. Owing to the relative scarcity of earlier studies, the level of evidence supporting acupuncture was previously low. In contrast, the number of high-quality studies on these techniques has gradually increased in recent years. To date, no study has systematically compared the efficacy and safety of different acupuncture modalities for intractable hiccups in a unified analytic framework. This study seeks to fill that gap by synthesizing direct and indirect evidence, providing a ranking of acupuncture interventions, and guiding future practice and research.

## 2 Materials and methods

This review was reported following the Preferred Reporting Items for Systematic Review and Meta-Analyses (PRISMA) for Network Meta-Analyses (NMA) (29) and was registered on the International Prospective Register of Systematic Reviews (PROSPERO) website (https://www.crd.york.ac.uk/prospero/) in January 2025 with the registration number CRD42025638314.

### 2.1 Inclusion and exclusion criteria

#### 2.1.1 Type of participants

Adult patients 18 years or above with a clinical diagnosis of intractable hiccups (hiccups lasting more than 48 h), regardless of gender, etiology, ethnic group, and severity.

#### 2.1.2 Types of interventions

The expected conventional acupuncture approaches include manual acupuncture (MA), moxibustion (Mox), electroacupuncture (EA), warm acupuncture (WA), auricular acupressure (AA), acupoint injection (AI), and transcutaneous electrical acupoint stimulation (TEAS), among others. The aforementioned acupuncture approaches may be used individually or in combination, regardless of the needling technique or acupoint selection. In addition, acupuncture combined with other therapies, such as Chinese herbal medicine (CHM) or conventional medications (CM), was also included within the scope of our analysis.

#### 2.1.3 Types of comparisons

Sham acupuncture (SA), CM, waiting list (WL), or acupuncture methods different from those used in the intervention group were considered control groups.

#### 2.1.4 Types of outcome indicators

The primary outcome is Total Effective Rate (TER), measured according to two common standards (30, 31), as cure rate + marked effective rate + remission rate. This will be judged by calculating clinical symptom scores, including parameters such as hiccup duration and frequency, etc. Secondary outcomes included the Hiccup Symptom Score (HSS), quality of life assessments (covering three items: diet, sleep, and mental status), and the incidence of adverse events.

#### 2.1.5 Type of study

Randomized controlled trials (RCTs) in Chinese and English were included.

#### 2.1.6 Exclusion criteria

a. Patients with hiccups lasting < 48 h.
b. Special acupuncture techniques such as scalp acupuncture, ocular acupuncture, wrist-ankle acupuncture, acupoint application, finger acupuncture, bee venom acupuncture, and bloodletting therapy.
c. Studies in which three or more types of therapies were used simultaneously.
d. Control groups that received the same type of acupuncture as the intervention group, differing only in needling techniques or selected acupoints.
e. Outcome indicators not related to IH.
f. Studies with unclear randomization methods or non-RCTs.
g. Studies with a treatment group sample size of 10 or fewer participants.

### 2.2 Information sources and search strategy

Two reviewers independently performed a comprehensive search of the following electronic databases: four English databases [PubMed, Embase, Cochrane Library, and Web of Science (WOS)] and four Chinese databases [China National Knowledge Infrastructure (CNKI), SinoMed, CQVIP, and Wanfang Database]. Additionally, four clinical trial registration platforms were searched: the World Health Organization International Clinical Trials Registry Platform (http://www.who.int/ictrp/en/), the Chinese Clinical Trial Registry (https://www.chictr.org.cn/), Clinical Trials.gov (https://www.clinicaltrials.govclinicaltrials.gov/) and the International Traditional Medicine Clinical Trial Registry (http://itmctr.ccebtcm.org.cn/) for acupuncture therapies for IH from January 2015 to January 2025. The following terms were imposed: (a) clinical conditions: hiccup, hiccough, singultus, etc.; (b) acupuncture therapy-related words: acupuncture, moxibustion, auricular acupressure, electroacupuncture, acupoint injection, etc.; and (c) trial type: an RCT. The terms “and” and “or” were combined between the search terms. Search strategies for these sources are shown in the Supplementary material.

### 2.3 Literature screening and data extraction

The exclusion of duplicate studies was performed in Endnote V.20 software, and then preliminary screening was performed by reading the title and abstract. After that, the full texts were further screened to exclude studies that did not meet the inclusion criteria. For data extraction, two researchers (LD and JC) separately conducted data extraction on the basis of the inclusion and exclusion criteria. If there was any disagreement, the third researcher (HZ) would make a final decision. The data extraction content included title, author, publication year and month, sample size, diagnostic criteria, interventions of treatment group and control group, dosage, course of treatment, and outcome indicators, among others.

### 2.4 Evaluation of the risk of bias

The quality evaluation was performed by two separate researchers (YZ and XT) using RCT Bias Risk Assessment Tool of the Cochrane System Review Manual Version 5.1.0, and a third researcher (HZ) would assist in judging the divergence between the two researchers. The evaluation items included random sequence generation, allocation concealment, blinding of patients and investigators, blinding of outcome evaluators, incomplete result data, selective reporting, and other biases.

### 2.5 Assessing certainty of the evidence

The Confidence in Network Meta-Analysis (CINeMA) system, a free and open-source CINeMA software (https://cinema.ispm.unibe.ch/), was used to assess credibility of results from network meta-analyses, which is based on the Grading of Recommendations Assessment, Development and Evaluation (GRADE)s and simplifies the evaluation process (32, 33). Six domains were evaluated, including: (a) within- study bias, (b) reporting bias, (c) indirectness, (d) imprecision, (e) heterogeneity, and (f) incoherence. Each domain was rated at 3 levels “no concerns,” “some concerns,” or “major concerns.”

### 2.6 Statistical analysis

Pairwise meta-analyses were conducted using Stata software V.17.0. The *I*^2^ statistic and *P-*value were used to identify and measure the heterogeneity among the studies. If *P* ≥ 0.05 and *I*^2^ < 50%, heterogeneity was considered not significant, and a fixed-effects model was used for meta-analysis. If *P* < 0.05 and *I*^2^ ≥ 50%, significant heterogeneity was assumed, and a random effects model was applied. In addition, for meta-analyses involving three-arm RCTs, the arms were separated into two-group comparisons for all possible pairwise combinations. For continuous variables, standardized mean difference (SMD) was used as the summary statistic, while risk ratio (RR) was used for dichotomous outcomes. Both effect measures were reported with 95% confidence intervals (CIs). A result was considered statistically significant when the 95% CI for RR did not include 1 or the 95% CI for SMD did not include 0.

The NMA was conducted within a frequentist framework using the network and mvmeta packages in Stata software. A network plot was generated to illustrate the comparisons between interventions, where the size of each node represented the sample size and the thickness of connecting lines indicated the number of studies contributing to each comparison. The surface under the cumulative ranking curve (SUCRA) was calculated to rank the probabilities of each intervention being the most effective. A SUCRA value closer to 100% indicates a higher probability of the intervention being the most effective. Additionally, publication bias and small-study effects were assessed using comparison-adjusted funnel plots. Finally, a two-outcomes cluster ranking plot was generated to illustrate the combined performance of each intervention in terms of both efficacy and safety.

## 3 Results

### 3.1 Literature screening results

**Figure 1:**
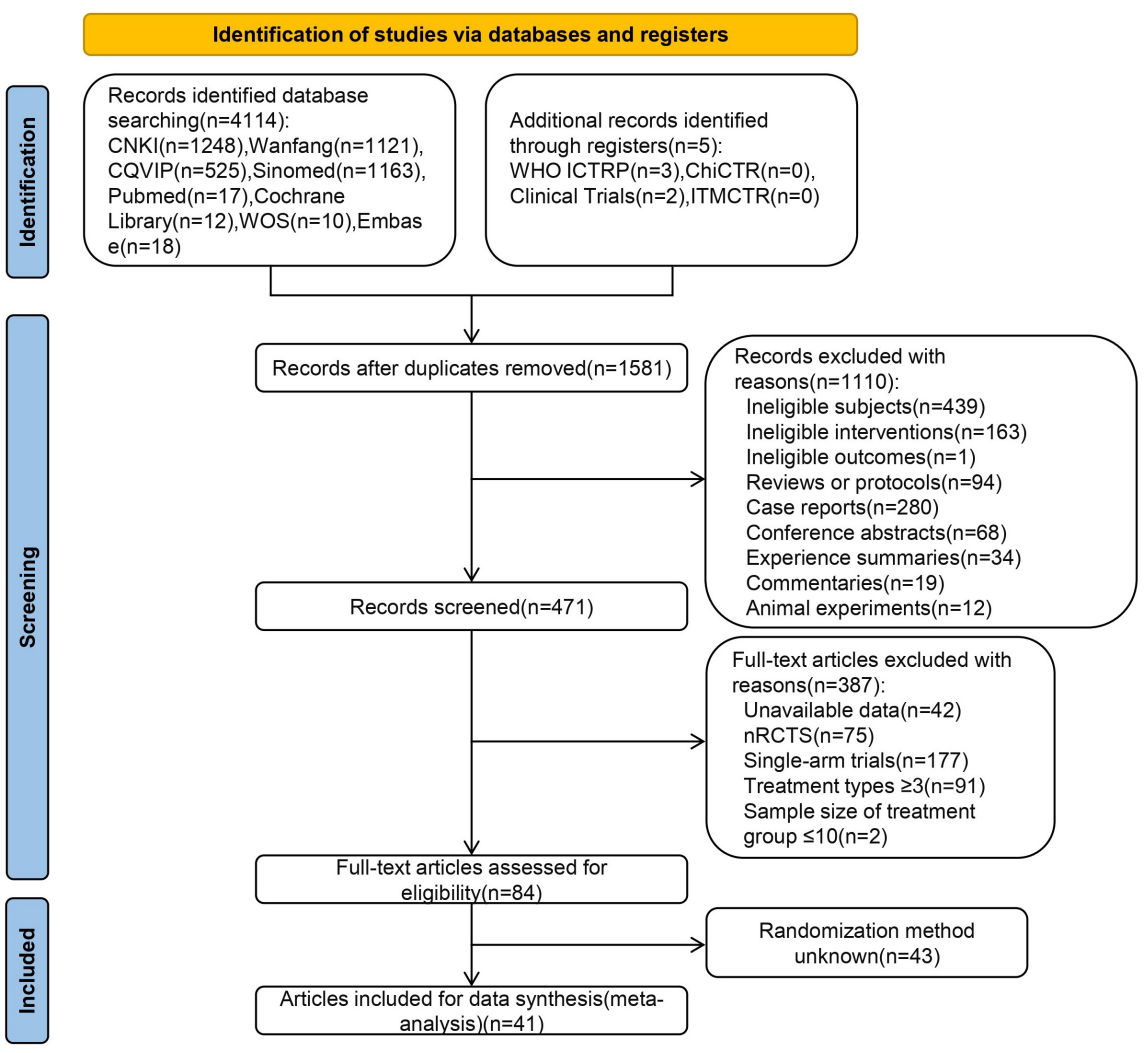
PRISMA flow diagram of the study selection process.

A total of 4,119 studies were retrieved, and 41 RCTs, including 3,060 patients, were ultimately included (15, 21–26, 34–67) after the preliminary screening and rescreening process. The literature screening process is shown in Figure 1.

### 3.2 Characteristics of the included studies

All 41 included studies were conducted in China and published in Chinese. Among them, only two were three-arm trials (24, 65), while the rest were two-arm trials. The five most frequently used treatment modalities in the intervention groups were MA+AI (21, 38, 49, 58, 62, 65, 67), MA (26, 40, 43, 44, 47, 48, 54), MA+CHM (34, 39, 45, 63, 64), EA (24, 36, 50, 53, 55), and MA+CM (15, 37, 57, 60, 66). In contrast, AI+CHM (35), EA+CM (59), AA (56), and TEAS+AI (42) were the least frequently used methods. Except for one study, all included trials reported total effective rate (TER). Eleven studies reported HSS (21, 23, 25, 26, 48, 51, 52, 57, 59, 62, 67); nine studies assessed diet, sleep, and mental status scores (23, 25, 38, 43, 44, 47, 48, 52, 62); and 17 studies reported the occurrence of adverse events (21, 23–26, 38, 43, 47, 49, 52, 53, 56, 61–63, 65, 67). Due to the very limited number of studies reporting outcomes on hiccup duration and frequency and the considerable heterogeneity in intervention types, substantial between-study heterogeneity was observed. Consequently, neither pairwise meta-analysis nor NMA was feasible for these outcomes, and no further description or analysis was performed. Detailed characteristics of the included studies are presented in Table 1.

**Table 1:** Characteristics of the included studies.

**Studies**	Primary diseases^*^	**Allocation ratio**	**Sample size**	**Age**	**Gender: M/F**	**(A)**	**(B)**	**(C)**	**Duration of treatment**	**Efficacy and safety criteria**	**Main results**
						**Treatment group**	**Control group I**	**Control group II**			
Wang 2024	Stroke	1:1	86	A:60.83 ± 5.82 B:61.37 ± 5.07	A:24/19 B:27/16	MA on Neiguan (PC 6), Cuanzhu (BL 2), with stimulation 1 × /day +(B)	AI [100 mg × 1/day dose of phenobarbital sodium injection on Zusanli (ST 36)]	/	7 days	1.Total effective rate; 2.Hiccup symptom score; 3.Adverse events	1.A>B; 2.A>B; 3.A=B
Xu 2023	Severe craniocerebral injury	1:1	60	A:47.86 ± 5.10 B:48.20 ± 4.98	A:19/11 B:18/12	MA on Zhongwan (RN 12), Neiguan (PC 6), Zusanli (ST 36), with stimulation 1 × /day +(B)	CM (10 mg × 1/day dose of metoclopramide hydrochloride injection for intramuscular injection)	/	3 days	Total effective rate	A>B
Li 2022	NR	1:1:1	120	A:49.68 ± 13.82 B:51.80 ± 11.80 C:53.93 ± 11.87	A:38/2 B:36/4 C:38/2	MA on Yingxiang (LI 20), Jingming (BL 1) Zusanli (ST 36), with stimulation 1 × /day +(C)	MA on Zhongwan (RN 12), Danzhong (RN 17), Geshu (BL 17), Neiguan (PC 6), Zusanli (ST 36), with stimulation 1 × /day	AI [1 ml/day dose of vitamin B6 and B12 injections on Zusanli (ST 36)]	3 days	1.Total effective rate; 2.Adverse events	1.A>B>C 2.A=B=C
Qin 2022	After cardiothoracic surgery	1:1:1	178	A:52.61 ± 15.54 B:53.22 ± 16.37 C:52.76 ± 15.54	A:47/23 B:46/22 C:28/12	EA on Neiguan (PC 6), Zusanli (ST 36), with continuous wave and low frequency stimulation 1 × /day	WA on Zhongkui (EX-UE4) with stimulation 1 × /day	CM (25–50 mg × 1/day dose of chlorpromazine hydrochloride injection for intramuscular injection)	5 days	1.Total effective rate; 2.Adverse events;	1.A>C>B 2.A=B>C
Fan 2021	After chemotherapy for lung carcinoma	1:1	63	A:59.22 ± 6.10 B:57.61 ± 7.58	A:27/5 B:25/6	AA on ear center (HX1), Shenmen (TF4), heart (CO15), with stimulation 3 × /day;MA on Neiguan (PC 6), Zusanli (ST 36), Cuanzhu (BL 2), with stimulation 1 × /day	CM (10 mg × 1/day dose of metoclopramide hydrochloride injection for intramuscular injection)	/	7 days	1.Total effective rate; 2.Hiccup symptom score 3.Dietary status score 4.Sleep status score 5.Mental status score 6.Adverse events	1.A>B; 2.A>B 3. A>B 4. A>B 5. A>B 6. A=B
Huang 2021	After radiotherapy or chemotherapy for malignant tumors	1:1	67	A:32–78 B:35–75	A:19/15 B:17/16	MA on Geshu (BL 17), Danzhong (RN 17), Tiantu (RN 22), Yifeng (SJ 17), Zhongwan (RN 12), Neiguan (PC 6), Zusanli (ST 36), Gongsun (SP 4), Cuanzhu (BL 2), with stimulation 1 × /day +(B)	CM (10 mg × 1/day dose of metoclopramide hydrochloride injection for intramuscular injection)	/	7 days	Total effective rate	A>B
Luo 2021	Stroke	1:1	86	A:44.13 ± 0.19 B:43.21 ± 0.15	A:20/23 B:21/22	TEAS on Neiguan (PC 6), Zhongwan (RN 12), Zusanli (ST 36), Geshu (BL 17), with 15 Hz stimulation 1 × /day; CHM (150 ml × 3/day dose of herbal decoction for oral)	CM [Two arms: (1) 5 mg × 3/day dose of metoclopramide tablets for oral. (2) 50 mg × 3/day dose of eperisone hydrochloride tablets for oral.]	/	7 days	1. Total effective rate; 2. Adverse events	1.A>B; 2.A>B
Ou yang 2021	NR	1:1	122	A:46.11 ± 3.09 B:46.74 ± 3.11	A:34/27 B:32/29	AI [10 mg/day dose of metoclopramide hydrochloride injection on Zusanli (ST 36)]+(B)	MA on Neiguan (PC 6), Zhongwan (RN 12), Cuanzhu (BL 2), Zusanli (ST 36), Shuigou (DU 26), with stimulation 1 × /day	/	2 weeks	1. Total effective rate 2. Hiccup symptom score 3. Dietary status score 4. Sleep status score 5. Mental status score 6. Adverse events	1.A>B; 2. A>B 3. A>B 4. A>B 5. A>B 6. A=B
Wang 2021	NR	1:1	86	A:42.50 ± 6.30 B:42.80 ± 5.60	A:21/22 B:23/20	MA on Tiantu (RN 22), Neiguan (PC 6), Zhongwan (RN 12), Zusanli (ST 36), Taichong (LR 3), with stimulation 1 × /day; CHM (200 ml × 1/day dose of herbal decoction for oral)	CM [(Two arms: (1) 0.6 mg × 2/day dose of Scopolamine injection for intramuscular injection; (2) 50 mg × 1/day dose of chlorpromazine hcl tablet for oral)]	/	5 days	1. Total effective rate 2. Adverse events	1. A>B 2. A=B
Yang 2021	After radiotherapy or chemotherapy for malignant tumors	1:1	60	A:54.0 ± 2.1 B:47.0 ± 1.9	A:16/14 B:17/13	MA on Cuanzhu (BL 2), Yuyao (EX-HN4), Neiguan (PC 6), Waiguan (SJ 5), Zusanli (ST 36), Yinlingquan (SP 9), with stimulation 1 × /day+(B)	CHM (200 ml × 2/day dose of herbal decoction for oral)	/	7 days	Total effective rate	A>B
Gan 2020	After transcatherter arterial chemoembolization (TACE) for hepatoma	1:1	82	A:49.23 ± 4.75 B:48.35 ± 5.12	A:30/11 B:28/13	MA on Dazhui (DU 14), Neiguan (PC 6), Danzhong (RN 17), Zhongwan (RN 12), Zusanli (ST 36), Shenque (RN 8), with stimulation 1 × /day+(B)	CM (10 mg × 2/day dose of metoclopramide hydrochloride injection for intramuscular injection)	/	7 days	Total effective rate	A>B
Liu 2020	NR	1:1	66	A:38.66 ± 10.15 B:37.72 ± 9.56	A:15/18 B:16/17	AI [5–10 mg/day dose of racemic anisodamine hydrochloride injection on cervical Jiaji (C3-C5)]+(B)	MA on Neiguan (PC 6), Zhongwan (RN 12), Zusanli (ST 36), Danzhong (RN 17), Geshu (BL 17), with stimulation 1 × /day	/	10 days	1.Total effective rate 2.Adverse events	1.A>B 2.A=B
Lu 2020	NR	1:1	86	A:54.24 ± 9.22 B:53.68 ± 9.43	A:21/22 B:22/21	EA on Geshu (BL 17), Weishu (BL 21), Neiguan (PC 6), Zusanli (ST 36), with dilatational wave stimulation 1 × /day+(B)	CM (50 mg × 3/day dose of itopride hydrochloride tablets for oral)	/	7 days	1.Total effective rate 2.Hiccup symptom score	1.A>B 2.A>B
Shen 2019	NR	1:1	60	A:38.7 ± 10.7 B:38.5 ± 10.9	A:18/12 B:17/13	MA on Geshu (BL 17), Neiguan (PC 6), Zhongwan (RN 12), Zusanli (ST 36), Tiantu (RN 22), Danzhong (RN 17), with stimulation 1 × /day	CM (10 mg × 2/day dose of Racemic anisodamine hydrochloride injection for intramuscular injection)	/	7 days	Total effective rate	A>B
Gao 2019	NR	1:1	60	A:43.4 ± 13.75 B:44.8 ± 13.87	A:17/13 B:16/14	EA on Cuanzhu (BL 2) with dilatational wave and high frequency stimulation 1 × /day	CM (5 mg × 2/day dose of Baclofen tablets for oral)	/	5 days	Total effective rate	A>B
Liu 2019 (1)	Traumatic brain injury, gastric surgery, stroke and fractured ribs	1:1	82	A:58.72 ± 10.65 B:59.01 ± 10.40	A:27/14 B:29/12	AA on ear center (HX1), Shenmen (TF4), stomach (CO4), sympathetic nerve (AH6a), subcortex (AT4), spleen (CO13), triple energizer (CO17), lung (CO14), with stimulation 3 × /day	CM (25 mg × 1/day dose of chlorpromazine hydrochloride injection for intramuscular injection)	/	12 days	1.Total effective rate 2.Adverse events	1.A>B 2.A>B
Liu 2019 (2)	Stroke	1:1	50	A:61.02 ± 3.16 B:60.77 ± 3.09	A:13/13 B:13/11	MA on Danzhong (RN 17), Neiguan (PC 6), Zhongwan (RN 12), Shuigou (DU 26), Cuanzhu (BL 2), Zusanli (ST 36), with stimulation 1 × /day	CM (25 mg × 3/day dose of chlorpromazine hydrochloride tablets for oral)	/	10 days	1.Total effective rate 2.Hiccup symptom score 3.Adverse events	1.A>B 2.A>B 3.A=B
Liu 2019 (3)	Stroke	1:1	60	A:57.22 ± 6.57 B:56.71 ± 6.09	A:19/11 B:17/13	MA on Cuanzhu (BL 2), Neiguan (PC 6), Zhongwan (RN 12), Zusanli (ST 36), Taichong (LR 3), with stimulation 1 × /day; AA on ear center (HX1), Shenmen (TF4), sympathetic nerve (AH6a), stomach (CO4), Liver (CO12), with stimulation 3 × /day	CM (5–10 mg × 3/day dose of baclofen tablets for oral)	/	6 days	1.Total effective rate 2.Hiccup symptom score 3.Dietary status score 4.Sleep status score 5.Mental status score 6.Adverse events	1.A>B 2.A>B 3.A>B 4.A=B 5.A>B 6.A=B
Wang 2019	Stroke	1:1	100	A:62.35 ± 4.2 B:59.6 ± 5.5	A:36/14 B:35/15	MA on Shuigou (DU 26), Cuanzhu (BL 2), Neiguan (PC 6), Danzhong (RN 17), Zhongwan (RN 12), Zusanli (ST 36), with stimulation 1 × /day+(B)	CM (10 mg × 2/day dose of metoclopramide hydrochloride injection for intramuscular injection)	/	7 days	Hiccup symptom score	A>B
Chen 2018	Stroke	1:1	68	A:60.79 ± 8.33 B:61.95 ± 6.72	A:21/14 B:18/15	AI (5 mg × 1/day dose of haloperidol injection on Zusanli (ST 36))	CM (10 mg × 2/day dose of haloperidol injection for intramuscular injection)	/	7 days	Total effective rate	A>B
Fan 2018	NR	1:1	112	A:48.6 ± 7.64 B:49.18 ± 7.56	A:35/21 B:37/19	MA on Sanyinjiao (SP 6), Danzhong (RN 17), Zusanli (ST 36), Taichong (LR 3), Neiguan (PC 6), with stimulation 1 × /day;AI [5 mg × 1/day dose of haloperidol injection on Zusanli (ST 36)]	CM (10 mg × 1/day dose of metoclopramide hydrochloride injection for intramuscular injection)	/	7 days	1.Total effective rate 2.Adverse events	1.A>B 2.A=B
Li 2018	NR	1:1	60	A:44.3 ± 13.95 B:45.9 ± 12.78	A:18/12 B:17/13	EA on Cuanzhu (BL 2), Tianding (LI 17), with dense wave stimulation 1 × /day	CM (5 mg × 2/day dose of baclofen tablets for oral)	/	5 days	Total effective rate	A>B
Wang 2018	Cerebral hemorrhage	1:1	120	A:68.5 ± 7.3 B:68.2 ± 7.9	A:56/4 B:52/8	MA on Shuigou (DU 26) with stimulation 1 × /day; AA on ear center (HX1), Shenmen (TF4), Stomach (CO4), with stimulation 3 × /day	MA on Zhongwan (RN 12), Shangwan (RN 13), Zusanli (ST 36), Geshu (BL 17), with stimulation 1 × /day	/	10 days	1.Total effective rate 2.Hiccup symptom score	1.A>B 2.A>B
Yang 2018	After radiotherapy or chemotherapy for malignant tumors	1:1	60	A:55.02 ± 6.53 B:54.68 ± 6.48	A:19/11 B:15/15	MA on Neiguan (PC 6), Zusanli (ST 36), Cuanzhu (BL 2), Danzhong (RN 17), Zhongwan (RN 12), with stimulation 1 × /day; AA on Ear center (HX1), Stomach (CO4), Sympathetic nerve (AH6a), Subcortex (AT4), with stimulation 3 × /day	CM (20 mg × 1/day dose of metoclopramide hydrochloride injection for intramuscular injection)	/	3 days	1.Total effective rate 2.Hiccup symptom score 3.Dietary status score 4.Sleep status score 5.Mental status score 6.Adverse events	1.A>B 2.A>B 3.A>B 4.A>B 5. A>B 6.A=B
Zhang 2018	After resection of hepatic neoplasms	1:1	39	A:47.1 ± 5.1 B:49.0 ± 5.5	A:12/8 B:12/7	EA on cervical Jiaji (C3-C5), with sparse wave stimulation 2 × /day	CM (Two arms: (1) 20 mg × 2/day dose of chlorpromazine hydrochloride injection for intramuscular injection; (2) 10 mg × 1/day dose of metoclopramide injection for intravenous infusion)	/	3 days	1. Total effective rate 2. Adverse events	1.A>B 2.A=B
Guo 2017	NR	1:1	60	A:53.40 ± 15.37 B:48.63 ± 10.00	A:12/18 B:14/16	MA on cervical Jiaji (C3–C5), with stimulation 1 × /day	CM (10 mg × 1/day dose of racemic anisodamine hydrochloride injection for intramuscular injection)	/	10 days	1.Total effective rate 2.Dietary status score 3.Sleep status score 4.Mental status score 5.Adverse events	1.A>B 2.A>B 3.A>B 4.A>B 5.A>B
Qiu 2017	Stroke	1:1	60	A:58.7 ± 10.76 B:61.57 ± 9.14	A:18/12 B:16/14	MA on Yangbai (GB 14), Yuyao (EX-HN4), Lianquan (RN 23), with stimulation 1 × /day	CM (10 mg × 1/day dose of racemic anisodamine hydrochloride injection for intramuscular injection)	/	10 days	1.Total effective rate 2.Hiccup symptom score 3.Dietary status score 4.Sleep status score 5.Mental status score	1.A>B 2.A>B 3.A>B 4.A>B 5.A>B
Zhong 2017	Stroke	1:1	120	A:66.0 ± 10.9 B:67.5 ± 11.1	A:28/32 B:19/41	MA on Danzhong (RN 17), Neiyingxiang (EX-HN9), Neiguan (PC 6), Gongsun (SP 4), Zusanli (ST 36), with stimulation 1 × /day;AI [10 mg × 1/day dose of metoclopramide hydrochloride injection on Neiguan (PC 6), Zusanli (ST 36)]	CM (Two arms: (1) 10 mg × 3/day dose of Baclofen tablets for oral; (2) 10 mg × 2/day dose of metoclopramide hydrochloride injection for Intramuscular injection)	/	7 days	1.Total effective rate 2.Hiccup symptom score 3.Adverse events	1.A>B 2.A>B 3.A=B
Cao 2016	NR	1:1	60	A:40.12 ± 12.11 B:40.78 ± 13.44	A:11/19 B:14/16	MA on Dingchuan (EX-B1), Geshu (BL 17), Feishu (BL 13), Danzhong (RN 17), Neiguan (PC 6), Cuanzhu (BL 2), with stimulation 1 × /day	CM (10 mg × 2/day dose of racemic anisodamine hydrochloride injection for intramuscular injection)	/	10 days	1.Total effective rate 2.Dietary status score 3.Sleep status score 4.Mental status score 5.Adverse events	1.A>B 2.A>B 3.A>B 4.A>B 5.A>B
Guo 2016	NR	1:1	60	A:51.30 ± 16.22 B:48.10 ± 17.26	A:17/13 B:16/14	MA on Yuyao (EX-HN4), Zhongkui (EX-UE4), with stimulation 1 × /day	CM (10 mg × 2/day dose of baclofen tablets for oral)	/	10 days	1.Total effective rate 2.Dietary status score 3.Sleep status score 4.Mental status score	1.A>B 2.A=B 3.A>B 4.A=B
Liu 2016	After chemotherapy for malignant tumors	1:1	48	A:54.5 B:52.2	A:16/8 B:15/9	MA on Neiguan (PC 6), Gongsun (SP 4), Zhongwan (RN 12), Danzhong (RN 17), with stimulation 1 × /day; CHM (200 ml × 2/day dose of herbal decoction for oral)	CM (20 mg × 1/day dose of metoclopramide hydrochloride injection for Intramuscular injection)	/	3 days	Total effective rate	A>B
Wu 2016	NR	1:1	60	A:51.42 ± 10.63 B:49.25 ± 11.09	A:18/12 B:17/13	AI (5 mg × 1/day dose of chlorpromazine hydrochloride injection on Zusanli (ST 36))	CM (20 mg × 1/day dose of racemic anisodamine hydrochloride injection for intramuscular injection)	/	3 days	Total effective rate	A>B
Gao 2015	NR	1:1	85	A:42.64 ± 10.52 B:42.35 ± 10.38	A:24/18 B:25/18	MA on Zhongwan (RN 12), Neiguan (PC 6), Zusanli (ST 36), Fenglong (ST 40), Taichong (LR 3), Shenque (RN 8), with stimulation 1 × /day+(B)	CHM (eight pills × 3/day dose of herbal pill for oral)	/	7 days	Total effective rate	A>B
Ke 2015	NR	1:1	76	A:45.9 ± 1.8 B:46.8 ± 1.3	A:21/17 B:19/19	AI [10 mg × 1/day dose of metoclopramide hydrochloride injection on Neiguan (PC 6)];CHM (150 ml × 2/day dose of herbal decoction for oral)	CM (10 mg × 1/day dose of metoclopramide hydrochloride injection for Intramuscular injection)	/	10 days	1.Total effective rate 2.Adverse events	1.A>B 2.A=B
Li 2015	Postoperative of oral cavity, abdomen, waist, and lower limbs	1:1	67	A:33 ± 12 B:33 ± 12	A:15/19 B:16/17	EA on Geshu (BL 17), Weishu (BL 21), with dilatational wave stimulation 1 × /day	CM [Two arms: (1) 10 mg × 2/day dose of metoclopramide hydrochloride injection for Intramuscular injection; (2) 10 mg × 2/day dose of Baclofen tablets for oral]	/	2 weeks	Total effective rate	A>B
Liao 2015	Postoperative of intracranial tumors	1:1	84	19–58	39/45	MA on Futu (LI 18), Neiguan (PC 6), Taichong (LR 3), with stimulation 1 × /day+(B)	CM (10 mg × 1/day dose of flunarizine hydrochloride capsules for oral)	/	2 weeks	Total effective rate	A>B
Lin 2015	NR	1:1	60	A:42.4 ± 6.93 B:41.7 ± 5.57	A:16/14 B:17/13	AI [10 mg × 1/day dose of metoclopramide hydrochloride injection on Neiguan (PC 6)]+(B)	MA on Cuanzhu (BL 2), Danzhong (RN 17), Zhongwan (RN 12), Neiguan (PC 6), Geshu (BL 17), Zusanli (ST 36), with stimulation 1 × /day	/	18 days	1.Total effective rate 2.Dietary status score 3.Sleep status score 4.Mental status score 5.Adverse events	1.A>B 2.A>B 3.A>B 4.A>B 5.A=B
Song 2015	Stroke	1:1	80	A:63.94 ± 8.36 B:62.31 ± 8.15	A:26/14 B:21/19	MA on Neiguan (PC 6), Zusanli (ST 36), Yintang (DU 29), Cuanzhu (BL 2) with stimulation 1 × /day; CHM (150 ml × 2/day dose of herbal decoction for oral)	CM (10 mg × 1/day dose of metoclopramide hydrochloride injection for Intramuscular injection)	/	7 days	Total effective rate	A>B
Zhang 2015 (1)	NR	1:1	30	A:67.4 ± 3.3 B:65.8 ± 3.2	A:8/7 B:6/9	MA on Zhongwan (RN 12), Xiawan (RN 10), Zusanli (ST 36), Shuigou (DU 26), Hegu (LI 4), Neiguan (PC 6), Tianshu (ST 25), Shangjuxu (ST 37), Taichong (LR 3), with stimulation 1 × /day	CM (10 mg × 1/day dose of metoclopramide hydrochloride injection for Intramuscular injection)	/	7 days	Total effective rate	A>B
Zhang 2015 (2)	NR	1:1	30	A:42.60 ± 12.56 B:43.87 ± 15.12	A:7/8 B:9/6	EA on Cuanzhu (BL 2), Neiguan (PC 6), Danzhong (RN 17), Zhongwan (RN 12), Zusanli (ST 36), with continuous wave stimulation 1 × /day;CHM (150 ml × 2/day dose of herbal decoction for oral)	CM (5 mg × 3/day dose of racemic anisodamine tablets for oral)	/	10 days	Total effective rate	A>B
Zhang 2015 (3)	Stroke	1:1	47	68.0 ± 7.2	29/18	TEAS on Neiguan (PC 6), Yifeng (SJ 17), with stimulation 1 × /day+(B)	AI (20 mg × 1/day dose of ritalin on Zusanli (ST 36))	/	3 days	Total effective rate	A>B

MA, manual acupuncture; CM, conventional medications; AI, acupoint injection; EA, electroacupuncture; AA, auricular acupressure; WA, warm acupuncture; CHM, Chinese herbal medicine; TEAS, transcutaneous electrical acupoint stimulation; NR, not reported.

^*^All studies reporting primary diseases mentioned that subjects received symptomatic underlying treatment for the primary diseases.

>The outcome or adverse events in the former is better than the latter by statistical analysis.

=There was no statistical difference between the former and the latter in this outcome.

### 3.3 Risk of bias among the included RCTs

**Figure 2:**
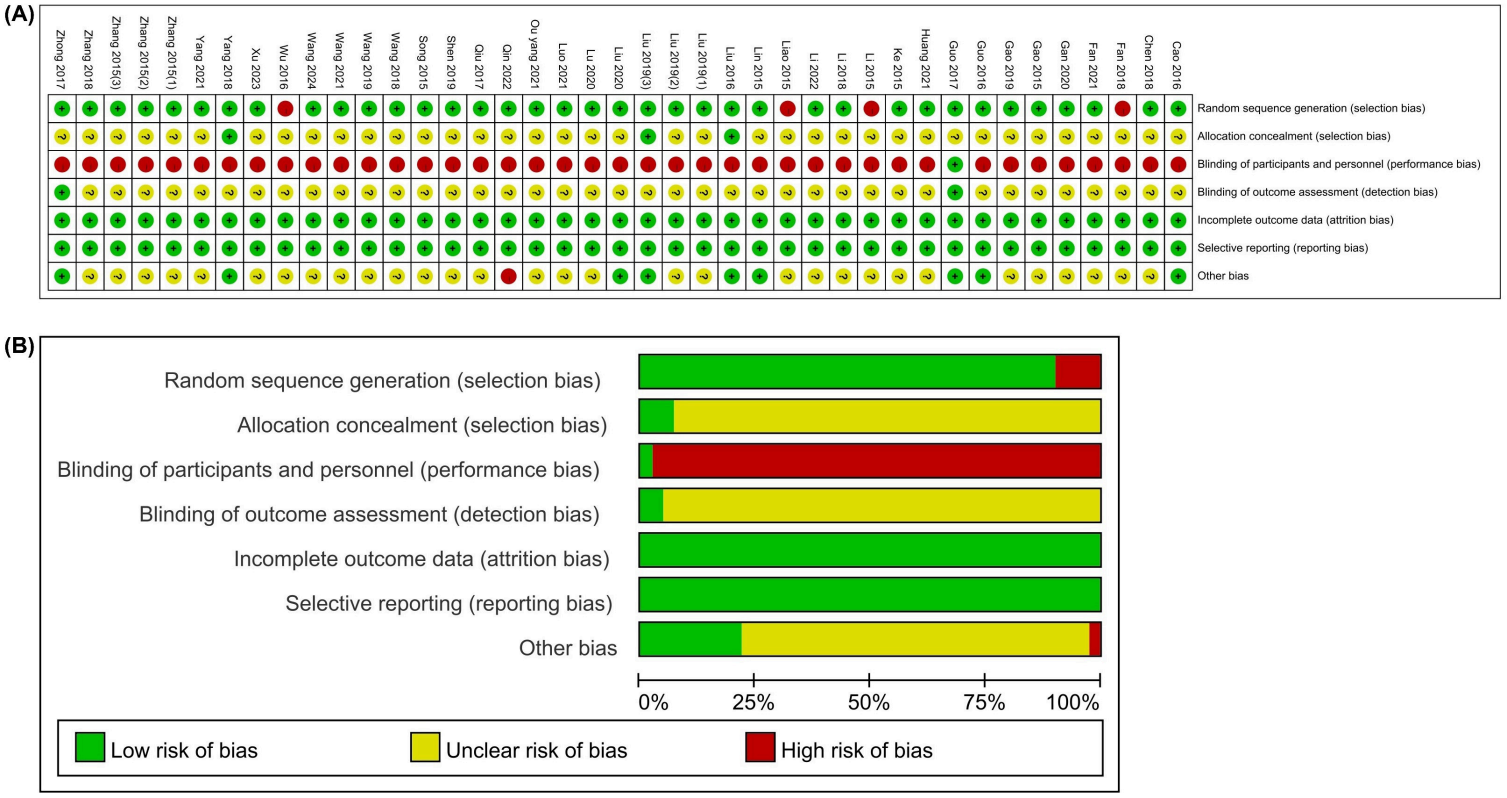
Risk of bias. **(A)** Risk of bias summary; **(B)** Risk of bias graph.

The risk of bias was assessed for each included study, and the results are presented in Figure 2. All studies explicitly described their randomization methods; four studies (36, 37, 46, 49) were rated as having a high risk of bias because group allocation was based on the order of patient visits, and one study (42) used coin tossing for randomization. The remaining studies adopted the random number table method. Three studies (25, 45, 52) reported using sealed, opaque envelopes for allocation concealment, whereas the remaining studies did not mention any method of concealment. Due to the nature of acupuncture procedure, blinding of practitioners was not feasible; thus, all studies were rated as having a high risk of performance bias. Except for two studies (21, 47), the blinding of outcome assessors and statisticians was not reported, resulting in an unclear risk of detection bias. All included studies had complete outcome data, and no selective reporting was identified. One study (24) was rated as high risk in the domain of other biases due to imbalanced baseline data.

### 3.4 Pairwise meta-analysis results

#### 3.4.1 Comparison of TER

A pairwise meta-analysis revealed that multiple acupuncture-related interventions were significantly more effective than CM or single treatments in improving TER among patients with IH. Specifically, MA+AI showed superior efficacy compared to both AI alone [two RCTs, RR = 1.20, 95% CI (1.05, 1.37)] and CM [two RCTs, RR = 1.35, 95% CI (1.18, 1.55)]. Similarly, MA+CM demonstrated a notable advantage over CM alone [four RCTs, RR = 1.35, 95% CI (1.21, 1.51)]. MA itself also showed enhanced effects when compared to CM [seven RCTs, RR = 1.27, 95% CI (1.15, 1.40)], and MA+CHM outperformed both CM [three RCTs, RR = 1.23, 95% CI (1.10, 1.38)] and CHM alone [two RCTs, RR = 1.47, 95% CI (1.23, 1.76)]. Moreover, EA was more effective than CM [five RCTs, RR = 1.28, 95% CI (1.17, 1.40)], and AI alone also showed better outcomes than CM [two RCTs, RR = 1.32, 95% CI (1.11, 1.58)]. In addition, other combined therapies, such as MA+AA, also showed superiority over CM [three RCTs, RR = 1.20, 95% CI (1.06, 1.36)], whereas TEAS+CHM was also more effective than CM [two RCTs, RR = 1.32, 95% CI (1.10, 1.58)]. Comparisons involving AA vs. CM, MA+AA vs. MA, and other combination therapies (such as AI+CHM, TEAS+AI, etc.) also demonstrated statistically significant advantages over their respective control groups, although some were based on a single study. Detailed results are presented in Table 2.

**Table 2:** Pairwise meta-analysis of total effective rate.

**Comparison categories**	**Number of studies**	***I* ^2^**	**RR (95% CI)**	***P*-value**
MA+AI vs. AI	2	0%	**1.20 (1.05, 1.37)**	**0.006**
MA+AI vs. CM	2	0%	**1.35 (1.18, 1.55)**	**< 0.0001**
MA+CM vs. CM	4	32%	**1.35 (1.21, 1.51)**	**< 0.00001**
MA+AI vs. MA	4	0%	**1.18 (1.09, 1.28)**	**< 0.0001**
AI vs. CM	2	0%	**1.32 (1.11, 1.58)**	**0.002**
AI+CHM vs. CM	1	–	**1.38 (1.10, 1.74)**	**0.005**
EA vs. WA	1	–	1.14 (1.00, 1.31)	0.05
EA vs. CM	5	77%	**1.28 (1.17, 1.40)**	**< 0.00001**
EA+CM vs. CM	1	–	1.28 (1.00, 1.62)	0.05
MA+AA vs. CM	3	0%	**1.20 (1.06, 1.36)**	**0.005**
MA+AA vs. MA	1	–	**1.11 (1.01, 1.23)**	**0.03**
AA vs. CM	1	–	**1.30 (1.07, 1.58)**	**0.009**
MA vs. CM	7	2%	**1.27 (1.15, 1.40)**	**< 0.00001**
TEAS+CHM vs. CM	2	68%	**1.32 (1.10, 1.58)**	**0.003**
TEAS+AI vs. AI	1	–	**1.54 (1.01, 2.34)**	**0.04**
MA+CHM vs. CM	3	0%	**1.23 (1.10, 1.38)**	**0.0004**
MA+CHM vs. CHM	2	0%	**1.47 (1.23, 1.76)**	**< 0.0001**
MA vs. AI	1	–	1.23 (0.93, 1.62)	=0.14
WA vs. CM	1	–	0.95 (0.81, 1.10)	=0.49

The bold font indicates a statistical difference.

#### 3.4.2 Comparison of HSS

A total of eight pairwise comparisons were reported on HSS. Compared with AI alone, MA+AI demonstrated superior symptom relief [one RCT, SMD = −0.86, 95% CI (−1.35, −0.37)], MA alone [one RCT, SMD = −1.20, 95% CI (−1.59, −0.81)], as well as CM [one RCT, SMD = −0.82, 95% CI (−1.16, −0.48)]. MA alone also significantly outperformed CM [two RCTs, SMD = −1.49, 95% CI (−2.14, −0.85)], and its combination with CM (MA+CM) produced even greater symptom improvement [one RCT, SMD = −2.04, 95% CI (−2.59, −1.49)]. Furthermore, EA+CM showed stronger efficacy than CM did [one RCT, SMD = −2.10, 95% CI (−2.79, −1.41)]. MA+AA also resulted in better symptom relief than CM [three RCTs, SMD = −1.32, 95% CI (−2.02, −0.61)] or MA alone [one RCT, SMD = −2.05, 95% CI (−2.71, −1.39)]. Detailed results are presented in Table 3.

**Table 3:** Pairwise meta-analysis of hiccup symptom score.

**Comparison categories**	**Number of studies**	***I* ^2^**	**SMD (95% CI)**	***P*-value**
MA+AI vs. AI	1	–	**−0.86 (−1.35**, **−0.37)**	**0.0005**
MA+AA vs. CM	3	52%	**−1.32 (−2.02**, **−0.61)**	**0.0003**
MA+AI vs. MA	1	–	**−1.20 (−1.59**, **−0.81)**	**< 0.00001**
EA+CM vs. CM	1	–	**−2.10 (−2.79**, **−1.41)**	**< 0.00001**
MA vs. CM	2	0%	**−1.49 (−2.14**, **−0.85)**	**< 0.00001**
MA+CM vs. CM	1	–	**−2.04 (−2.59**, **−1.49)**	**< 0.00001**
MA+AA vs. MA	1	–	**−2.05 (−2.71**, **−1.39)**	**< 0.00001**
MA+AI vs. CM	1	–	**−0.82 (−1.16**, **−0.48)**	**< 0.00001**

The bold font indicates a statistical difference.

#### 3.4.3 Comparison of quality-of-life scores

In terms of quality-of-life scores, three intervention comparisons were conducted here. The results revealed that MA+AI demonstrated significantly greater improvements across the three domains compared with MA alone (two RCTs). Specifically, improvements were observed in dietary status [SMD = 0.85, 95% CI (0.65, 1.06)], sleep status [SMD = 0.52, 95% CI (0.31, 0.72)], and mental status [SMD = 0.47, 95% CI (0.29, 0.65)]. Similarly, MA+AA was superior to CM in all three domains (three RCTs), with significant improvements in dietary status [SMD = 0.57, 95% CI (0.37, 0.78)], sleep status [SMD = 0.37, 95% CI (0.19, 0.55)], and mental status [SMD = 0.60, 95% CI (0.42, 0.78)]. In addition, MA alone also had more favorable effects than CM did (four RCTs). Improvements were observed in dietary status [SMD = 0.70, 95% CI (0.02, 1.38)], sleep status [SMD = 0.47, 95% CI (0.26, 0.69)], and mental status [SMD = 0.68, 95% CI (0.47, 0.90)], suggesting the potential of MA to alleviate relevant symptoms. Detailed results are presented in Table 4.

**Table 4:** Pairwise meta-analysis of dietary/sleep/mental status score.

**Comparison categories**	**Number of studies**	**Dietary status score**			**Sleep status score**			**Mental status score**		
		*I* ^2^	**SMD (95% CI)**	***P*** **-value**	*I* ^2^	**SMD (95% CI)**	***P*** **-value**	*I* ^2^	**SMD (95% CI)**	***P*** **-value**
MA+AI vs. MA	2	0%	**0.85 (0.65, 1.06)**	**< 0.00001**	0%	**0.52 (0.31, 0.72)**	**< 0.00001**	54%	**0.47 (0.29, 0.65)**	**< 0.00001**
MA+AA vs. CM	3	8%	**0.57 (0.37, 0.78)**	**< 0.00001**	77%	**0.37 (0.19, 0.55)**	**< 0.0001**	37%	**0.60 (0.42, 0.78)**	**< 0.00001**
MA vs. CM	4	89%	**0.70 (0.02, 1.38)**	**< 0.00001**	43%	**0.47 (0.26, 0.69)**	**< 0.0001**	51%	**0.68 (0.47, 0.90)**	**< 0.00001**

The bold font indicates a statistical difference.

### 3.5 Network meta-analysis results

#### 3.5.1 Network plot for multiple interventions

**Figure 3:**
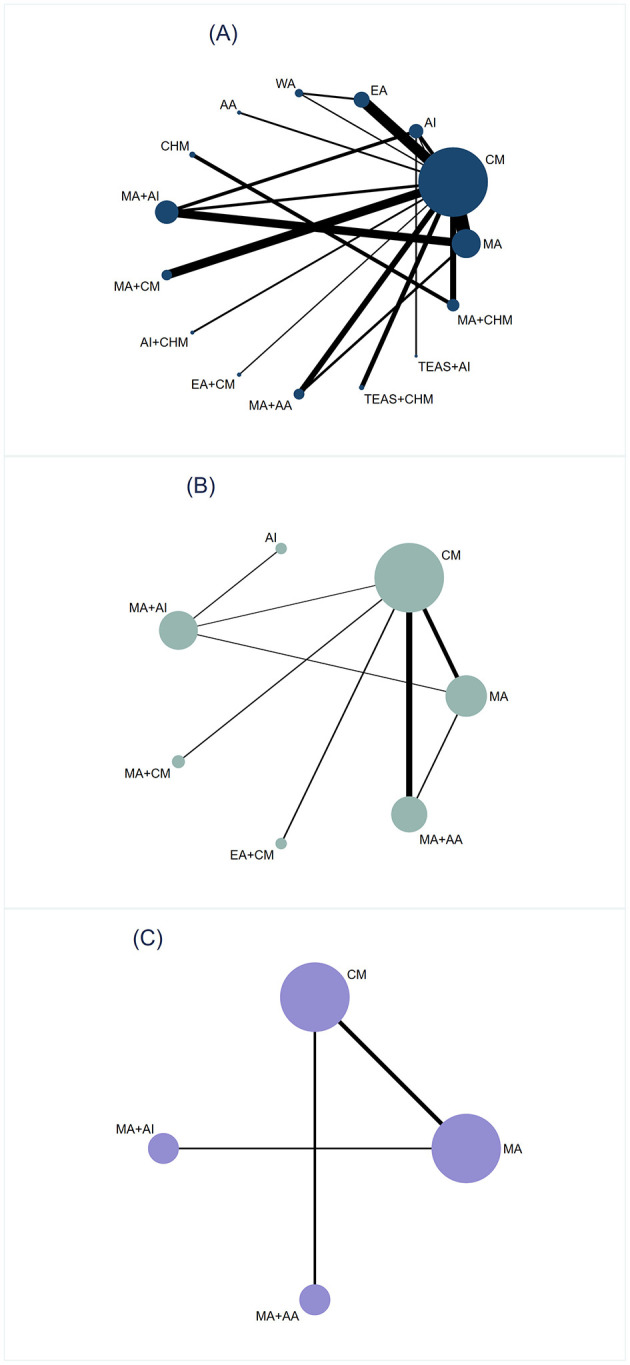
Network plot. **(A)** Total effective rate. **(B)** Hiccup symptom score. **(C)** Dietary/Sleep/Mental status score.

In Figure 3, the thickness of the line is positively correlated with the two intervention methods, whereas the size of the points is proportional to the weight of the sample size in the intervention. A total of 40 studies reported the TER, involving 19 different interventions and 2,960 participants, forming six closed loops. Eleven studies reported HSS, covering seven treatment strategies and 927 participants, forming two closed loops. Nine studies assessed quality-of-life in three domains—dietary, sleep, and mental status—across four treatment approaches and a total of 605 participants; no closed loop was formed.

#### 3.5.2 Inconsistency test

**Figure 4:**
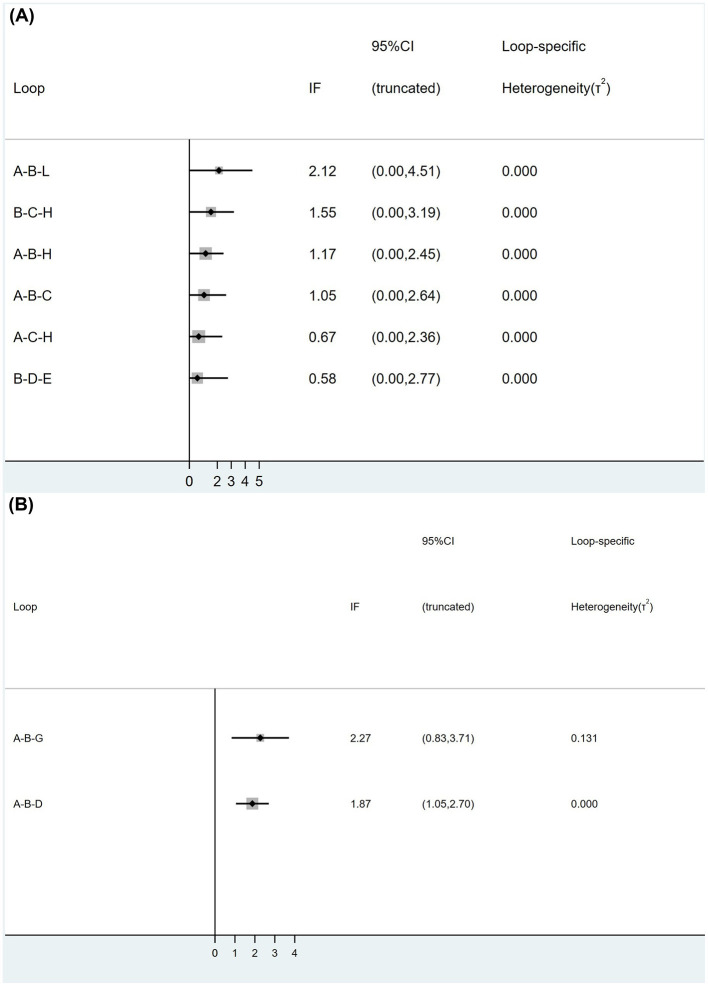
The inconsistency test conducted on the closed loops formed by the three intervention points of TER **(A)** and HSS **(B)**.

The inconsistency test was conducted on the six triangular closed loops formed by the study of TER, and the results showed that Loop (A-B-L) *P* = 0.082, Loop (B-C-H) *P* = 0.065, Loop (A-B-H) *P* = 0.073, Loop (A-B-C) *P* = 0.191, Loop (A-C-H) *P* = 0.435, Loop (B-D-E) *P* = 0.607. All *P*-values were >0.05, indicating good consistency between direct and indirect evidence. Inconsistency test was performed on the two closed loops formed by the study of reporting HSS, and the results showed that: Loop (A-B-G) *P* = 0.002, Loop (A-B-D) *P* = 0.000. All *P*-values were < 0.05, revealing statistically significant inconsistency, suggesting potential discrepancies between direct and indirect comparisons. Detailed results are presented in Figure 4. In order to identify the source of the inconsistency, we rechecked the data and coding, and conducted a node splitting analysis. We found that MA vs. CM (A-B) contributed the most to the inconsistency (*P* < 0.001). Since the studies reporting dietary, sleep, and mental status scores did not form any closed loops, inconsistency tests could not be performed.

#### 3.5.3 NMA results of TER

In the comparison of single interventions, MA was significantly more effective than WA [RR = 4.38, 95% CI (1.29, 14.84)] and CHM [RR = 6.08, 95% CI (1.30, 28.49)] were effective; EA outperformed both WA [RR = 8.48, 95% CI (2.45, 29.35)] and CHM [RR = 11.77, 95% CI (2.28, 60.85)] were effective; and AA was also superior to WA [RR = 0.10, 95% CI (0.01, 0.70)] and CHM [RR = 13.72, 95% CI (1.59, 118.34)]. Overall, WA and CHM consistently showed lower RR values compared with other treatments, suggesting that their therapeutic efficacy may be relatively limited. Moreover, combined interventions demonstrated greater advantages over single interventions, likely due to synergistic effects. MA+AI was superior to MA [RR = 0.34, 95% (0.19, 0.62)], AI [RR = 0.27, 95% (0.13, 0.54)], WA [RR = 0.08, 95% (0.02, 0.28)], and CHM [RR = 0.06, 95% (0.01, 0.27)]; MA+CM outperformed AI [RR = 0.32, 95% (0.11, 0.95)], WA [RR = 0.09, 95% (0.02, 0.38)], and CHM [RR = 0.07, 95% (0.01, 0.36)]; and TEAS+AI was more effective than AI [RR = 0.23, 95% (0.06, 0.89)], WA [RR = 0.07, 95% (0.01, 0.44)], and CHM [RR = 0.05, 95% (0.01, 0.40)]. However, EA+CM showed no advantage over any other intervention except CM [RR = 2.98, 95% (1.02, 8.70)]. Compared with the control intervention CM, all the other treatments, except WA and CHM, showed significant efficacy. Detailed results are presented in Table 5.

**Table 5:** Network meta-analysis of total effective rate [RR (95% CI)].

**MA**														
1.28 (0.65, 2.55)	**AI**													
0.52 (0.21, 1.26)	0.40 (0.15, 1.10)	**EA**												
**4.38 (1.29, 14.84)**	3.41 (0.92, 12.65)	**8.48 (2.45, 29.35)**	**WA**											
0.44 (0.08, 2.32)	0.35 (0.06, 1.93)	0.86 (0.15, 4.92)	**0.10 (0.01, 0.70)**	**AA**										
**6.08 (1.30, 28.49)**	4.74 (0.94, 23.85)	**11.77 (2.28, 60.85)**	1.39 (0.22, 8.77)	**13.72 (1.59, 118.34)**	**CHM**									
**0.34 (0.19, 0.62)**	**0.27 (0.13, 0.54)**	0.66 (0.26, 1.70)	**0.08 (0.02, 0.28)**	0.77 (0.14, 4.15)	**0.06 (0.01, 0.27)**	**MA+AI**								
0.41 (0.15, 1.09)	**0.32 (0.11, 0.95)**	0.78 (0.25, 2.44)	**0.09 (0.02, 0.38)**	0.91 (0.15, 5.52)	**0.07 (0.01, 0.36)**	1.18 (0.42, 3.33)	**MA+CM**							
0.38 (0.07, 1.99)	0.30 (0.05, 1.66)	0.74 (0.13, 4.23)	**0.09 (0.01, 0.60)**	0.86 (0.09, 8.04)	**0.06 (0.01, 0.54)**	1.11 (0.21, 5.99)	0.94 (0.16, 5.68)	**AI+CHM**						
1.07 (0.33, 3.46)	0.83 (0.23, 2.96)	2.06 (0.56, 7.60)	0.24 (0.05, 1.15)	2.40 (0.36, 16.22)	0.18 (0.03, 1.08)	3.11 (0.92, 10.52)	2.63 (0.66, 10.38)	2.79 (0.41, 18.84)	**EA+CM**					
0.66 (0.26, 1.68)	0.51 (0.17, 1.49)	1.27 (0.41, 3.91)	**0.15 (0.04, 0.61)**	1.48 (0.25, 8.88)	**0.11 (0.02, 0.59)**	1.91 (0.70, 5.25)	1.62 (0.48, 5.40)	1.72 (0.29, 10.31)	0.62 (0.16, 2.42)	**MA+AA**				
0.58 (0.16, 2.06)	0.45 (0.12, 1.75)	1.12 (0.28, 4.48)	**0.13 (0.03, 0.67)**	1.30 (0.18, 9.32)	**0.09 (0.01, 0.62)**	1.68 (0.45, 6.24)	1.42 (0.33, 6.10)	1.51 (0.21, 10.83)	0.54 (0.11, 2.66)	0.88 (0.21, 3.75)	**TEAS+CHM**			
0.29 (0.06, 1.35)	**0.23 (0.06, 0.89)**	0.57 (0.10, 3.08)	**0.07 (0.01, 0.44)**	0.66 (0.07, 5.93)	**0.05 (0.01, 0.40)**	0.86 (0.19, 3.96)	0.72 (0.13, 4.15)	0.77 (0.09, 6.88)	0.28 (0.04, 1.77)	0.45 (0.08, 2.53)	0.51 (0.07, 3.48)	**TEAS+AI**		
0.63 (0.22, 1.83)	0.49 (0.15, 1.58)	1.23 (0.37, 4.08)	**0.14 (0.03, 0.63)**	1.43 (0.23, 9.00)	**0.10 (0.03, 0.32)**	1.85 (0.61, 5.60)	1.56 (0.44, 5.60)	1.66 (0.26, 10.46)	0.59 (0.14, 2.48)	0.97 (0.27, 3.44)	1.10 (0.24, 4.97)	2.16 (0.36, 12.94)	**MA+CHM**	
**3.17 (1.95, 5.16)**	**2.47 (1.25, 4.88)**	**6.14 (2.92, 12.89)**	0.72 (0.24, 2.21)	**7.15 (1.47, 34.71)**	0.52 (0.12, 2.26)	**9.26 (5.19, 16.50)**	**7.82 (3.31, 18.46)**	**8.31 (1.71, 40.32)**	**2.98 (1.02, 8.70)**	**4.84 (2.07, 11.30)**	**5.50 (1.70, 17.82)**	**10.81 (2.36, 49.43)**	**5.01 (1.94, 12.89)**	**CM**

The bold font indicates a statistical difference.

#### 3.5.4 NMA results of HSS

Comparisons among acupuncture-related therapies revealed that only MA+AI was significantly more effective than MA [SMD = 0.78, 95% CI (0.10, 1.47)], and MA+AA outperformed MA [SMD = 0.66, 95% CI (0.05, 1.27)]; other comparisons did not reach statistical significance. Except for MA and AI, all other interventions showed greater benefit compared to CM, specifically: MA+AI [SMD = −1.18, 95% CI (−1.86, −0.49)], MA+CM [SMD = −1.44, 95% CI (−2.36, −0.52)], EA+CM [SMD = −1.28, 95% CI (−2.21, −0.35)], and MA+AA [SMD = −1.05, 95% CI (−1.55, −0.56)]. Detailed results are presented in Table 6.

**Table 6:** Network meta-analysis of hiccup symptom score [SMD (95% CI)].

**MA**						
0.04 (−1.10, 1.19)	**AI**					
**0.78 (0.10, 1.47)**	0.74 (−0.18, 1.66)	**MA+AI**				
1.05 (−0.01, 2.11)	1.00 (−0.46, 2.47)	0.26 (−0.88, 1.41)	**MA+CM**			
0.88 (−0.19, 1.95)	0.84 (−0.63, 2.31)	0.10 (−1.05, 1.25)	−0.16 (−1.47, 1.14)	**EA+CM**		
**0.66 (0.05, 1.27)**	0.62 (−0.60, 1.83)	−0.12 (−0.92, 0.67)	−0.39 (−1.43, 0.66)	−0.22 (−1.28, 0.83)	**MA+AA**	
−0.39 (−0.92, 0.14)	−0.44 (−1.58, 0.71)	**−1.18 (−1.86**, **−0.49)**	**−1.44 (−2.36**, **−0.52)**	**−1.28 (−2.21**, **−0.35)**	**−1.05 (−1.55**, **−0.56)**	**CM**

The bold font indicates a statistical difference.

#### 3.5.5 NMA results of quality-of-life scores

As shown in Table 7, only four intervention strategies were included in the analysis of quality-of-life scores. In terms of improving dietary status, both MA [SMD = 0.92, 95% CI (0.13, 1.71)] and MA+AI [SMD = 1.95, 95% CI (0.60, 3.30)] were significantly more effective than CM, while the difference between MA+AA and CM was not statistically significant. For sleep status, MA [SMD = −0.52, 95% CI (−0.92, −0.12)], MA+AI [SMD = −1.22, 95% CI (−1.88, −0.55)], and MA+AA [SMD = −0.58, 95% CI (−1.05, −0.12)] all showed superior effects compared to CM. Regarding improvements in mental status (Table 8), CM showed no advantage over MA [SMD = 0.75, 95% CI (0.46, 1.03)], MA+AI [SMD = 1.54, 95% CI (1.10, 1.99)], or MA+AA [SMD = 1.00, 95% CI (0.67, 1.34)]. Notably, MA combined with AI showed significantly greater improvements in both sleep status [SMD = 0.69, 95% CI (0.16, 1.23)] and mental status [SMD = −0.80, 95% CI (−1.14, −0.45)] compared to MA alone, indicating a potential synergistic effect.

**Table 7:** Network meta-analysis of dietary status score (lower left quadrant) and sleep status score [right upper quadrant; SMD (95% CI)].

**MA**	**0.69 (0.16, 1.23)**	**0.06 (−0.55, 0.67)**	**−0.52 (−0.92, −0.12)**
−1.03 (−2.13, 0.06)	**MA+AI**	−0.63 (−1.44, 0.18)	**−1.22 (−1.88**, **−0.55)**
0.06 (−1.15, 1.26)	1.09 (−0.54, 2.72)	**MA+AA**	**−0.58 (−1.05**, **−0.12)**
**0.92 (0.13, 1.71)**	**1.95 (0.60, 3.30)**	0.86 (−0.05, 1.77)	**CM**

The bold font indicates a statistical difference.

**Table 8:** Network meta-analysis of mental status score [SMD (95% CI)].

**MA**			
**−0.80 (−1.14**, **−0.45)**	**MA+AI**		
−0.26 (−0.70, 0.18)	0.54 (−0.02, 1.10)	**MA+AA**	
**0.75 (0.46, 1.03)**	**1.54 (1.10, 1.99)**	**1.00 (0.67, 1.34)**	**CM**

The bold font indicates a statistical difference.

#### 3.5.6 SUCRA probability ranking results

**Figure 5:**
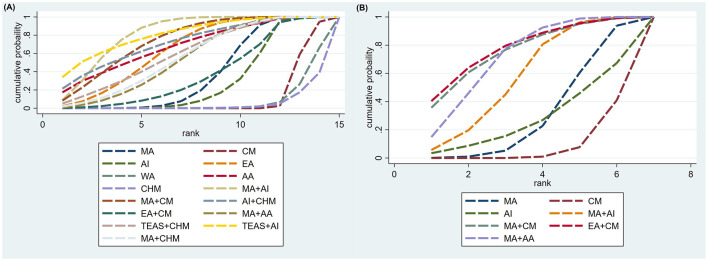
SUCRA plot. **(A)** TER; **(B)** HSS.

**Figure 6:**
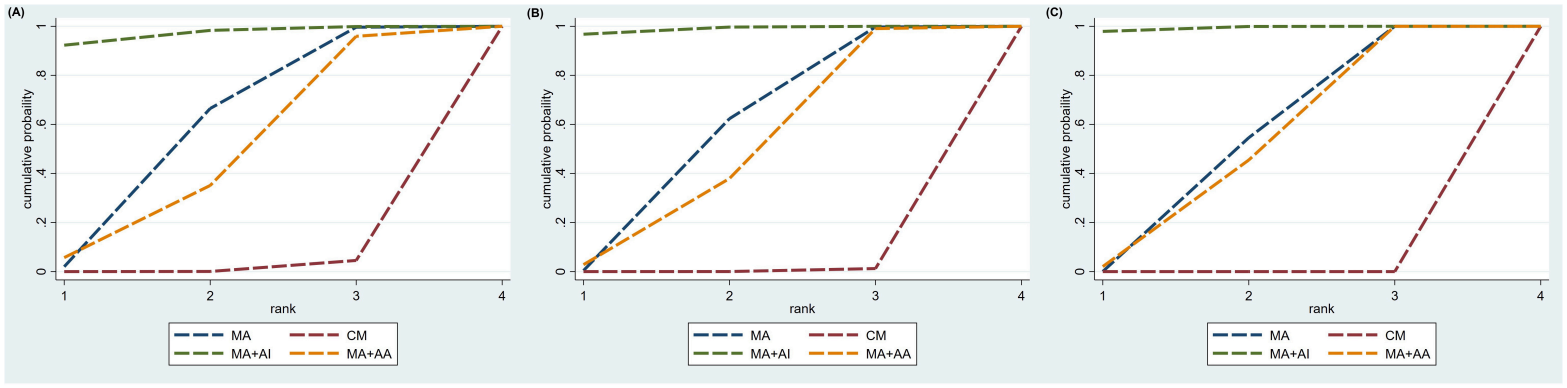
SUCRA plot. **(A)** Dietary status score; **(B)** sleep status score; **(C)** mental status score.

First, the top four interventions with the highest TER were all combination therapies: MA+AI (82.2%), TEAS+AI (81.5%), MA+CM (74.8%), and AI+CHM (72.9%). Among the monotherapies, the most effective were AA (69.1%), EA (65.4%), and MA (37.9%), while CM ranked the lowest (11.2%). Second, in terms of reducing HSS, the interventions ranked from most to least effective were: EA+CM (77.9%), MA+CM (75.8%), MA+AA (71.6%), MA+AI (57.8%), MA (30.6%), AI (28.0%), and CM (8.3%). Finally, the interventions ranked identically across improvements in dietary, sleep, and mental status scores: MA+AI > MA > MA+AA > CM. Detailed results are presented in Figures 5, 6 and Table 9.

**Table 9:** Ranking of interventions based on SUCRA value (%).

**Treatment**	**TER**		**HSS**		**Dietary status score**		**Sleep status score**		**Mental status score**	
	**SUCRA (%)**	**Rank**	**SUCRA (%)**	**Rank**	**SUCRA (%)**	**Rank**	**SUCRA (%)**	**Rank**	**SUCRA (%)**	**Rank**
MA	37.9	11	30.6	5	56.0	2	54.2	2	51.5	2
CM	11.2	13	8.3	7	1.5	4	0.4	4	0.0	4
AI	29.9	12	28.0	6	–	–	–	–	–	–
EA	65.4	6	–	–	–	–	–	–	–	–
WA	7.4	14	–	–	–	–	–	–	–	–
AA	69.1	5	–	–	–	–	–	–	–	–
CHM	4.7	15	–	–	–	–	–	–	–	–
MA+AI	82.2	1	57.8	4	96.8	1	98.8	1	99.3	1
MA+CM	74.8	3	75.8	2	–	–	–	–	–	–
AI+CHM	72.9	4	–	–	–	–	–	–	–	–
EA+CM	38.4	10	77.9	1	–	–	–	–	–	–
MA+AA	56.0	9	71.6	3	45.6	3	46.6	3	49.2	3
TEAS+CHM	61.2	7	–	–	–	–	–	–	–	–
TEAS+AI	81.5	2	–	–	–	–	–	–	–	–
MA+CHM	57.4	8	–	–	–	–	–	–	–	–

#### 3.5.7 Publication bias

**Figure 7:**
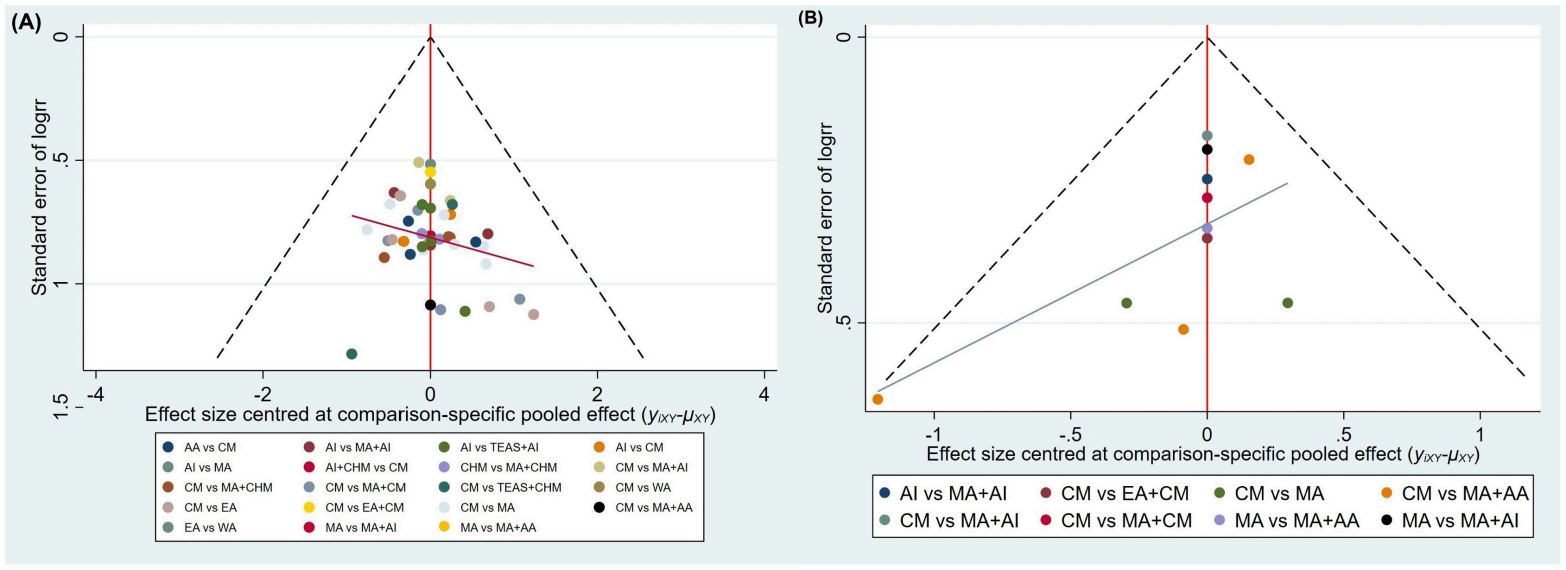
The comparison-adjusted funnel plots showed no major asymmetry for TER **(A)**, suggesting a low likelihood of substantial publication bias. In contrast, the HSS **(B)** funnel plot displayed visible asymmetry and a left-tilted regression line, indicating potential small-study effects and possible overestimation of treatment efficacy. Given the limited number of HSS studies and greater instability in their distribution.

Comparison-adjusted funnel plots were constructed for the primary outcome TER and the secondary outcome HSS to assess the potential for publication bias. As shown in Figure 7A, studies related to TER were generally symmetrically distributed around the vertical axis in the central part of the inverted funnel, suggesting a low risk of publication bias. A few studies appeared at the bottom of the plot, which may be attributed to smaller sample sizes. Figure 7B shows that some studies reporting HSS were clustered along the vertical line, while the rest were scattered asymmetrically, indicating the potential presence of publication bias or small-study effects within the research network. Given that fewer than 10 studies reported quality-of-life scores, funnel plot analysis was not conducted due to insufficient power to detect asymmetry.

### 3.6 The confidence assessment results of NMA

The CINeMA system (https://cinema.ispm.unibe.ch/) based on the GRADE framework was used to classify the confidence in the results of NMA, which six domains was evaluated, including: (a) within- study bias, (b) reporting bias, (c) indirectness, (d) imprecision, (e) heterogeneity, and (f) incoherence. Among the 19 confidence ratings for the comparisons, six were rated as “very low,” seven as “low,” only six as “moderate,” and none of the comparisons received a “high” rating. The detailed assessment content is presented in Table 10.

**Table 10:** The confidence assessment result of NMA.

**Comparison**	**Number of studies**	**Within-study bias**	**Reporting bias**	**Indirectness**	**Imprecision**	**Heterogeneity**	**Incoherence**	**Confidence rating**	**Reason(s) for downgrading**
AA:CM	1	Some concerns	Low risk	No concerns	No concerns	No concerns	Major concerns	Low	[“Within-study bias,” “incoherence”]
AI:CM	2	Some concerns	Low risk	No concerns	No concerns	Major concerns	Major concerns	Very low	[“Within-study bias,” “heterogeneity,” “incoherence”]
AI:MA	1	Some concerns	Low risk	No concerns	Major concerns	No concerns	No concerns	Low	[“Within-study bias,” “imprecision”]
AI:MA+AI	2	Some concerns	Low risk	No concerns	No concerns	No concerns	No concerns	Moderate	[“Within-study bias”]
AI:TEAS+AI	1	Some concerns	Low risk	No concerns	No concerns	Major concerns	Major concerns	Very low	[“Within-study bias,” “heterogeneity,” “incoherence”]
AI+CHM:CM	1	Some concerns	Low risk	No concerns	No concerns	No concerns	Major concerns	Low	[“Within-study bias,” “incoherence”]
CHM:MA+CHM	2	Some concerns	Low risk	No concerns	No concerns	No concerns	Major concerns	Low	[“Within-study bias,” “incoherence”]
CM:EA	5	Major concerns	Low risk	No concerns	No concerns	No concerns	Major concerns	Very low	[“Within-study bias,” “incoherence”]
CM:EA+CM	1	Some concerns	Low risk	No concerns	Major concerns	No concerns	Major concerns	Very low	[“Within-study bias,” “imprecision,” “incoherence”]
CM:MA	7	Some concerns	Low risk	No concerns	No concerns	No concerns	No concerns	Moderate	[“Within-study bias”]
CM:MA+AA	3	Some concerns	Low risk	No concerns	No concerns	No concerns	No concerns	Moderate	[“Within-study bias”]
CM:MA+AI	2	Some concerns	Low risk	No concerns	No concerns	No concerns	No concerns	Moderate	[“Within-study bias”]
CM:MA+CHM	3	Some concerns	Low risk	No concerns	No concerns	No concerns	Major concerns	Low	[“Within-study bias,” “incoherence”]
CM:MA+CM	4	Some concerns	Low risk	No concerns	No concerns	No concerns	Major concerns	Low	[“Within-study bias,” “incoherence”]
CM:TEAS+CHM	2	Some concerns	Low risk	No concerns	No concerns	No concerns	Major concerns	Low	[“Within-study bias,” “incoherence”]
CM:WA	1	Major concerns	Low risk	No concerns	Major concerns	No concerns	Major concerns	Very low	[“Within-study bias,” “imprecision,” “incoherence”]
EA:WA	1	Major concerns	Low risk	No concerns	No concerns	Major concerns	Major concerns	Very low	[“Within-study bias,” “heterogeneity,” “incoherence”]
MA:MA+AA	1	Some concerns	Low risk	No concerns	Major concerns	No concerns	No concerns	Moderate	[“Within-study bias,” “imprecision”]
MA:MA+AI	4	Some concerns	Low risk	No concerns	No concerns	No concerns	No concerns	Moderate	[“Within-study bias”]

### 3.7 Safety

The occurrence of adverse events was mentioned in 17 studies. Overall, the number of adverse events reported in the treatment groups receiving various acupuncture-related therapies was significantly lower than that in the CM group, and no serious adverse reactions were observed, indicating the safety of acupuncture-based interventions (Table 11).

**Table 11:** Adverse events of included studies.

**Studies**	**Comparisons**	**Adverse events**	
		**Treatment group**	**Control group**
Wang 2024	MA+AI vs. AI	Two cases of rash, one case of general weakness	One case of rash, two cases of headache, one case of constipation, one case of general weakness
Li 2022	MA+AI vs. MA vs. AI	One case of nasal swelling and pain	MA: one case of needle sickness; AI: None
Qin 2022	EA vs. WA vs. CM	One case of inappetence, one case of acupoint swelling and pain	CM: two cases of inappetence, two cases of drowsiness, four cases of palpitation, two case of acupoint swelling and pain; WA: one case of acupoint swelling and pain
Fan 2021	MA+AA vs. CM	None	Two cases of severe fatigue, constipation and thirst
Luo 2021	TEAS+CHM vs. CM	None	Two cases of drowsiness, three cases of inappetence, two cases of constipation, three cases of fatigue, one case of dizziness
Ou yang 2021	MA+AI vs. MA	None	None
Wang 2021	MA+CHM vs. CM	None	None
Liu 2019 (1)	AA vs. CM	One case of dizziness	Three cases of dizziness, two cases of inappetence, one case of drowsiness
Liu 2019 (2)	MA vs. CM	One case of rash, two cases of diarrhea and constipation, one case of dizziness	Two cases of rash, two cases of diarrhea and constipation, one case of dizziness, two cases of mild sleep dysfunction
Liu 2019 (3)	MA+AA vs. CM	One case of subcutaneous hematoma	Two cases of dizziness
Fan 2018	MA+AI vs. CM	None	None
Yang 2018	MA+AA vs. CM	None	None
Zhang 2018	EA vs. CM	None	None
Guo 2017	MA vs. CM	One case of drowsiness, one case of thirst	Two cases of drowsiness, three cases of fatigue, one case of diarrhea, five cases of thirst, one case of blurred vision
Zhong 2017	MA+AI vs. CM	One case of fatigue	One case of fatigue, two cases of dizziness, one case of drowsiness
Cao 2016	MA vs. CM	One case of fatigue, one case of drowsiness	Five case of fatigue, three case of drowsiness, four cases of dizziness, three cases of diarrhea, two cases of eyes dry
Lin 2015	MA+AI vs. MA	None	None

### 3.8 Cluster ranking plot for multiple interventions

**Figure 8:**
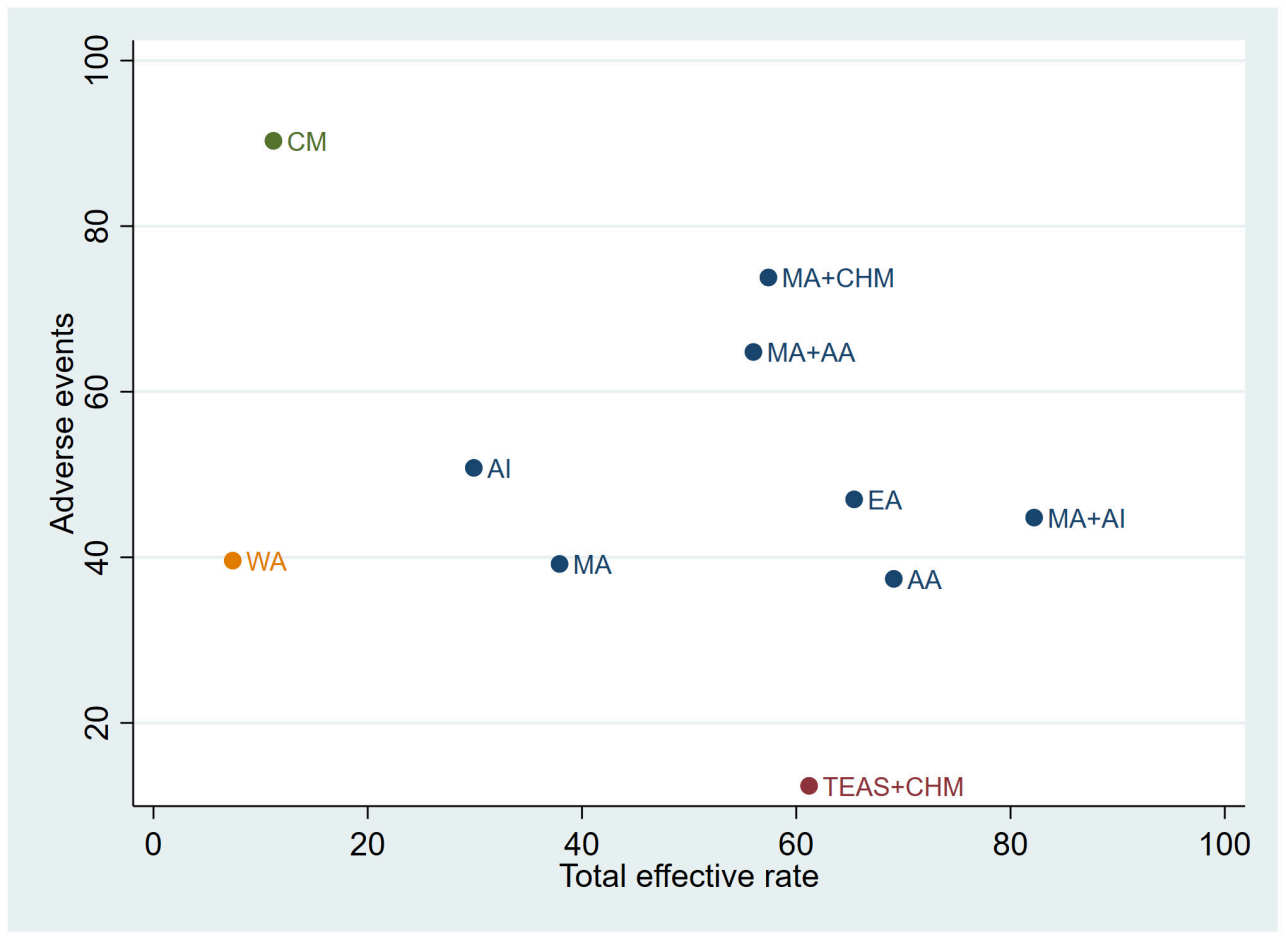
The cluster analysis, which demonstrates that the intervention measures have both good overall efficiency and safety performance.

A cluster analysis based on the SUCRA values for TER and the incidence of adverse events was conducted to evaluate different interventions (Figure 8). A total of 10 interventions were included, which were grouped into four distinct clusters, each represented by a different color. Interventions such as MA+AI, AA, and EA demonstrated both high efficacy and good safety, making them the most favorable options. Although TEAS+CHM showed the best safety profile, its efficacy was inferior compared to the aforementioned interventions. MA+AA and MA+CHM exhibited relatively balanced profiles, with moderate-to-high efficacy and acceptable safety.

## 4 Discussion

### 4.1 Main results

Our study assessed the effects of various acupuncture techniques—applied individually or in combination—on five key outcomes in patients with IH: TER, HSS, and quality-of-life parameters, including dietary, sleep, and mental status. TER, a commonly reported composite outcome in TCM research, is defined as the proportion of patients achieving predefined clinical improvement or cure (30). Its binary nature facilitates direct comparisons across interventions and reflects changes across multiple symptoms (68). HSS provides a continuous measure of symptom severity, encompassing frequency, intensity, and duration, allowing detection of partial yet clinically meaningful improvements (23). The impact of IH on patients' quality of life can be devastating (69). For example, cancer patients often avoid eating to prevent hiccup episodes, leading to malnutrition and delayed recovery (69–71). Hiccup-related sleep disturbances, such as doubled sleep latency, may induce chronic psychological stress and further frustrate patients with comorbid speech disorders (72, 73). This burden may worsen underlying conditions or provoke psychiatric symptoms, including poststroke depression (73, 74). Therefore, dietary, sleep and mental status were included as patient-centered secondary outcomes. These dimensions have been highlighted in prior meta-analyses on cancer-related and poststroke hiccups, showing that quality-of-life improvements often mirror symptom relief (27, 28).

The clinical benefits of acupuncture for IH observed in our NMA can be reconciled with mechanistic data at both peripheral and central levels. Hiccups are generated by a reflex arc involving vagal and phrenic afferents, brainstem networks (including the nucleus tractus solitarius and adjacent respiratory centers), and phrenic efferents (3–7). Acupuncture, particularly MA and EA, can regulate this reflex arc through multisite neural activation. Experimental studies have shown that EA improves gastric motility and heart rate variability via vagovagal modulation, reflecting enhanced parasympathetic tone and inhibition of abnormal reflex excitability (75, 76). At the spinal and supraspinal levels, EA has been demonstrated to modulate neuronal activity in specific autonomic circuits controlling gastrointestinal function (77) and to reduce aberrant excitability in reflex-related interneurons through TRPV1-dependent mechanisms (78). AA involves attaching Vaccaria seeds or magnetic beads to specific auricular points with appropriate pressure stimulation (79). The ear is richly innervated and vascularized, with dense neural networks—particularly involving the auricular branch of the vagus nerve—concentrated in the cavum conchae and triangular fossa (80). Gao et al. (81) suggest that stimulating these auricular points may activate the auricular branch of the vagus nerve, which, through the auricle–central–visceral axis, modulates visceral activities such as gastrointestinal motility to suppress hiccups. AI combines the mechanical stimulation of acupuncture with the pharmacologic effects of injected substances, producing synergistic modulation of local microcirculation and neurochemical signaling, which may further contribute to reflex inhibition (82). Compared with intramuscular injection, AI has been shown to reduce hiccup frequency and alleviate symptoms more effectively under the same dosage and medication conditions (22, 83). From a physiological perspective, by activating cutaneous afferents and downstream central networks, acupuncture can modulate autonomic outflow, inflammatory signaling, and neuroendocrine responses (84, 85). Together, these findings support that acupuncture alleviates IH through an integrated neurophysiological mechanism involving vagovagal reflex modulation, spinal inhibitory network activation, and neurochemical homeostasis restoration.

Our study yielded relatively reliable results by directly and indirectly comparing the effectiveness of different acupuncture interventions for IH. Regarding the TER, pairwise meta-analysis revealed that among monotherapies, AI, MA, EA, and AA were all more effective than CM. Moreover, combination therapies were generally superior to single modalities—for example, MA+AI was more effective than both AI and MA; MA+AA outperformed MA; TEAS+AI was superior to AI; and acupuncture combined with CM (e.g., MA+CM, EA+CM) was more effective than CM alone. According to the SUCRA-based probability ranking, MA+AI was the most effective, followed by TEAS+AI, MA+CM, and AI+CHM. In terms of reducing HSS, pairwise meta-analyses indicated that EA+CM was superior to CM, and MA—whether used alone or in combination with AI, AA, or CM—was consistently more effective than CM. The NMA results ranked EA+CM as the most effective for lowering HSS, followed by MA and MA+AA. In terms of quality of life, both MA monotherapy and its combination with AA demonstrated statistically significant improvements. NMA results further suggested that MA+AI was the most effective intervention across all quality-of-life domains, regardless of diet, sleep, or mental state. Overall, although effectiveness varied across different outcomes, MA+AI consistently ranked first for both TER and quality-of-life improvements. While its incidence of adverse events was not the lowest, MA+AI appears to be the optimal choice for treating IH, given its superior efficacy and acceptable safety profile. It should be noted that MA+AI was the most frequently reported intervention among studies evaluating TER (*n* = 8), and it generally outperformed the control group. This “high connection density” enhanced the statistical stability, amplified the therapeutic effect trend after aggregation, and thus formed the optimal result in the network comparison.

### 4.2 Limitations

Our review has important limitations that should be considered with caution. First, all included trials were conducted in China and published in Chinese, introducing substantial language and regional publication bias. Therefore, the results of this NMA chiefly reflect the clinical context and practice patterns in China. This concentrated geographic and linguistic source not only elevates the risk of selective reporting but also limits the external validity and generalizability of our findings to global clinical settings, where practitioner training, acupuncture techniques, standard care, and patient expectations may differ. Second, based on the confidence assessment results, all of the evidence levels range from “very low” to “moderate,” with none achieving a “high” level of certainty. This indicates that the overall quality of evidence supporting the comparative effectiveness of acupuncture interventions for IH remains suboptimal. The downgrading was primarily due to the high or unclear risk of bias, inconsistency among studies, and imprecision of the estimated effects. As acknowledged, blinding of acupuncturists and participants was not feasible because of the nature of acupuncture procedure. Moreover, most studies did not adequately describe allocation concealment or blinding of outcome assessors, resulting in potential performance and detection bias. These methodological weaknesses may have inflated the observed treatment effects. Participants who were aware of receiving active treatment may have experienced expectancy-related improvements, especially for subjective outcomes. Likewise, the absence of assessor blinding could introduce observer bias, further amplifying efficacy estimates. Since all outcomes in this study were based on self-reports or assessments by unblinded evaluators, the results are particularly vulnerable to such biases. In addition, the use of non-standardized outcome definitions also affects comparability across studies and may reduce the generalizability of the findings. Third, the node-splitting analysis revealed significant inconsistency in the comparisons involving MA vs. CM (*P* < 0.001). This may be attributed to the fact that MA–CM formed the core linkage of several triangular loops, amplifying any discrepancy between direct and indirect evidence. Clinically, MA and CM generally show relatively modest differences in efficacy, whereas the indirect comparisons often involve combination therapies with stronger effects (such as MA+AI or MA+AA). This imbalance in treatment magnitude may have exaggerated inconsistencies in the overall network. Additionally, variations in pharmacological regimens (Liu 2019 (2): chlorpromazine/Qiu 2017: anisodamine), acupoint selection, and needling manipulation across studies may further contribute to this divergence, thereby reducing the confidence in network estimates for the HSS. Fourth, none of the included studies involved a WL or SA, which restricts our ability to determine the specific efficacy of acupuncture interventions beyond nonspecific or placebo effects. Consequently, while the included trials demonstrate comparative effectiveness among active treatments, the absolute magnitude of benefit attributable solely to acupuncture remains unclear. Fifth, since the variety of acupuncture interventions, there are inconsistencies in acupoint selection, treatment duration and frequency, which may lead to heterogeneity. In addition, we suggest that acupuncture-related RCTs should be designed according to the CONSORT (86) and STRICTA (87) to control the quality of future studies.

## 5 Conclusion

This network meta-analysis highlights the effectiveness and safety of acupuncture in treating intractable hiccups within the clinical setting of China, particularly in terms of total effective rate, symptom relief, and quality of life improvement. After a comprehensive comparison of 15 different interventions, the results indicate that combination therapies outperform monotherapies. Manual acupuncture combined with acupoint injection emerged as the most effective approach for improving both total effective rate and quality of life, and given its favorable safety profile, it may represent the optimal treatment option for intractable hiccups. Nonetheless, the statistically generated favorable bias and numerical dominance that amplify the observed trend of efficacy should also be taken into account. On the other hand, electroacupuncture combined with conventional medications was the most effective for reducing hiccup symptoms. Of course, these findings are based on the premise that the primary diseases of intractable hiccups can be accurately diagnosed and appropriately treated. In clinical practice, the choice of therapy should also be tailored to the patient's specific condition. Given the limited number of existing studies and their suboptimal methodological quality, coupled with geographical and linguistic constraints, more rigorously designed mechanism studies and international, strictly conducted randomized controlled trials are needed to validate our findings.

## Data Availability

All data analyzed in this systematic review and network meta-analysis are derived from previously published studies. The full search strategies, list of included studies, and extracted data are available within the Supplementary material of this article. No new primary data were generated.
