# Supplementary material for: Effectiveness and safety of acupuncture therapies for intractable hiccups: a systematic review and network meta-analysis
Source: Front Med (Lausanne). 2025 Nov 19;12:1676850. doi: 10.3389/fmed.2025.1676850 (PMC12672884; doi:10.3389/fmed.2025.1676850)
Supplement: Supplementary file 1 [file Table_1.docx]

**Search checklist of each database**

**Pubmed**

#1 "Hiccup"[Mesh]

#2 "Hiccup"[Mesh] OR ”Hiccup*”[tw] OR Hiccough[tw] OR ”Hiccough*” [tw] OR Singultus[tw]

#3 #1 OR #2

#4 "Acupuncture Therapy"[Mesh]

#5 "Acupuncture Therapy"[Mesh] OR "Acupuncture Therapy"[tw] OR acupuncture[tw] OR Moxibustion[tw] OR “Acupuncture, Ear” [tw] OR "Auricular acupressure"[tw] OR Electroacupuncture[tw] OR “Meridian*”[tw] OR “Acupuncture Point*”[tw] OR “Acupoint*”[tw] OR "Warm needle"[tw] OR "Manual-acupuncture"[tw] OR "Transcutaneous electrical acupoint stimulation"[tw] OR "TEAS"[tw]

#6 #4 OR #5

#7 "Randomized Controlled Trial" [Publication Type] OR "Random Allocation"[Mesh] OR "Randomized Controlled Trials as Topic"[Mesh] OR "Controlled Clinical Trial" [Publication Type]

#8 Clinical[tw] OR Trial[tw] OR Clinical trial[tw] OR Random[tw]

#9 #7 OR #8

#10 #3 AND #6 AND #9

**Results:40**

**Cochrane Library**

ID Search Hits

#1 MeSH descriptor: [Hiccup] explode all trees

#2 (“Hiccup”):ti,ab,kw OR (“Hiccups”):ti,ab,kw OR (Hiccough):ti,ab,kw OR (“Hiccoughs” ):ti,ab,kw OR (Singultus):ti,ab,kw

#3 #1 OR #2

#4 MeSH descriptor: [Acupuncture Therapy] explode all trees

#5 ("Acupuncture Therapy"):ti,ab,kw OR (acupuncture):ti,ab,kw OR (Moxibustion):ti,ab,kw OR (“Acupuncture, Ear” ):ti,ab,kw OR ("Auricular acupressure"):ti,ab,kw OR (Electroacupuncture):ti,ab,kw OR (“Meridian”):ti,ab,kw OR (“Acupuncture Point”):ti,ab,kw OR (“Acupoint”):ti,ab,kw OR ("Auricular acupressure"):ti,ab,kw OR ("Warm needle"):ti,ab,kw OR ("Manual-acupuncture"):ti,ab,kw OR ("Transcutaneous electrical acupoint stimulation"):ti,ab,kw OR ("TEAS"):ti,ab,kw

#6 #4 OR #5

#7 (Randomized Controlled Trial):pt (Word variations have been searched)

#8 ("Randomized Controlled Trial"):ti,ab,kw OR ("Random Allocation"):ti,ab,kw OR (Clinical):ti,ab,kw OR (Trial):ti,ab,kw OR (“Clinical trial”):ti,ab,kw OR (Random):ti,ab,kw OR (Randomly):ti,ab,kw OR (Randomized):ti,ab,kw OR (Randomization):ti,ab,kw OR (Randomised):ti,ab,kw

#9 #7 OR #8

#10 #3 AND #6 AND #9 in Trials

**Results:29**

**Web of Science**

Query #1

"Hiccup*" OR "Hiccough*" OR "Singultus" (Topic) and Preprint Citation Index (Exclude – Database)

Query #2

"Acupuncture Therapy" OR "Acupuncture Therapy" OR “acupuncture” OR “Moxibustion” OR “Acupuncture, Ear” OR "Auricular acupressure" OR “Electroacupuncture” OR “Meridian*” OR “Acupuncture Point*” OR “Acupoint*” OR "Auricular acupressure" OR "Warm needle" OR "Manual-acupuncture" OR "Transcutaneous electrical acupoint stimulation" OR "TEAS" (Topic) and Preprint Citation Index (Exclude – Database)

Query #3

"Randomized Controlled Trial" OR "Random Allocation" OR "Randomized Controlled Trials as Topic" OR "Controlled Clinical Trial" OR “Clinical” OR “Trial” OR “Clinical trial” OR “Random*” (Topic) and Preprint Citation Index (Exclude – Database)

Query #4

#1 AND #2 AND #3 and Preprint Citation Index (Exclude – Database)

**Results:24**

**Embase**

#1

('hiccup*':ti,ab,kw OR 'hiccough*':ti,ab,kw OR 'Singultus':ti,ab,kw OR 'hiccup'/exp) AND [2015-2025]/py

#2

('acupuncture therapy':ti,ab,kw OR 'acupuncture':ti,ab,kw OR 'moxibustion':ti,ab,kw OR 'acupuncture, ear':ti,ab,kw OR 'electroacupuncture':ti,ab,kw OR 'meridian*':ti,ab,kw OR 'acupuncture point*':ti,ab,kw OR 'acupoint*':ti,ab,kw OR 'Auricular acupressure':ti,ab,kw OR 'warm needle':ti,ab,kw OR 'manual-acupuncture':ti,ab,kw OR 'transcutaneous electrical acupoint stimulation':ti,ab,kw OR 'teas':ti,ab,kw OR 'acupuncture therapy'/exp) AND [2015-2025]/py

#3

('randomized controlled trial':ti,ab,kw OR 'random allocation':ti,ab,kw OR 'randomized controlled trials as topic':ti,ab,kw OR 'controlled clinical trial':ti,ab,kw OR 'clinical':ti,ab,kw OR 'trial':ti,ab,kw OR 'clinical trial':ti,ab,kw OR 'random*':ti,ab,kw OR 'randomized controlled trial'/exp) AND [2015-2025]/py

**Results:39**

**CNKI**

(主题:顽固性呃逆) AND (篇关摘:顽固性呃逆 + 持续性呃逆 + 难治性呃逆 + 重症呃逆 + 膈肌痉挛 + 打嗝 + 哕(精确)) AND (主题:针刺) AND (篇关摘:针刺 + 针灸 + 艾灸 + 毫针 + 耳穴 + 耳针 + 电针 + 手针 + 温针 + 穴位注射 + 火针 + 拔罐 + 经皮穴位电刺激(精确)) AND (主题:随机对照试验) AND (篇关摘:随机对照试验 + 随机对照研究 + 随机 + 对照 + 临床观察 + 疗效观察 + 效果分析 + 临床研究(精确)) AND 发表时间:2015-*

**Results:1248**

**Wanfang**

(主题:(顽固性呃逆) and 题名或关键词:(顽固性呃 or 持续性呃逆 or 难治性呃逆 or 重症呃逆 or 膈肌痉挛 or 打嗝 or 哕) and 主题:(针刺) and 题名或关键词:(针刺 or 针灸 or 艾灸 or 毫针 or 耳穴 or 耳针 or 电针 or 温针 or 穴位注射 or 火针 or 拔罐 or 经皮穴位电刺激) and 主题:(随机对照试验) and 题名或关键词:(随机对照试验 or 随机对照研究 or 随机 or 对照 or 临床观察 or 疗效观察 or 效果分析 or 临床研究)) and 发表时间:2015-*

**Results:1121**

**ChongqingVIP**

(((((((题名或关键词=顽固性呃逆 OR 题名或关键词=持续性呃逆) OR 题名或关键词=难治性呃逆) OR 题名或关键词=重症呃逆) OR 题名或关键词=膈肌痉挛) OR 题名或关键词=打嗝) OR 题名或关键词=哕) AND (((((((((((((题名或关键词=针刺 OR 题名或关键词=针灸) OR 题名或关键词=艾灸) OR 题名或关键词=毫针) OR 题名或关键词=耳穴) OR 题名或关键词=耳针) OR 题名或关键词=电针) OR 题名或关键词=手针) OR 题名或关键词=温针) OR 题名或关键词=穴位注射) OR 题名或关键词=火针) OR 题名或关键词=拔罐) OR 题名或关键词=经皮电刺激) AND ((((((((题名或关键词=随机对照试验) OR 题名或关键词=随机对照研究) OR 题名或关键词=随机) OR 题名或关键词=对照) OR 题名或关键词=临床观察) OR 题名或关键词=疗效观察) OR 题名或关键词=效果分析) OR 题名或关键词=临床研究) AND (years:[2015 TO 2025])

**Results:525**

**CBM**

(((((((( "顽固性呃逆"[常用字段] OR "持续性呃逆"[常用字段] OR "难治性呃逆"[常用字段] OR "重症呃逆"[常用字段] OR "膈肌痉挛"[常用字段] OR "打嗝"[常用字段] OR "哕"[常用字段])) AND 2015-2025[日期])) AND (((( "针刺"[常用字段:智能] OR "针灸"[常用字段:智能] OR "艾灸"[常用字段:智能] OR "毫针"[常用字段:智能] OR "耳穴"[常用字段:智能] OR "耳针"[常用字段:智能] OR "电针"[常用字段:智能] OR "温针"[常用字段:智能] OR "穴位注射"[常用字段:智能] OR "火针"[常用字段:智能] OR "拔罐"[常用字段:智能] OR "经皮穴位电刺激"[常用字段:智能])) AND 2015-2025[日期])))) AND (((( "随机对照试验"[常用字段:智能] OR "随机对照研究"[常用字段:智能] OR "随机"[常用字段:智能] OR "对照"[常用字段:智能] OR "临床观察"[常用字段:智能] OR "疗效观察"[常用字段:智能] OR "效果分析"[常用字段:智能] OR "临床研究"[常用字段:智能])) AND 2015-2025[日期])))) AND 2015-2025[日期] **Results:1163**
